# Molecular Mechanisms of Phosphate Use Efficiency in *Arabidopsis* via *Penicillium olsonii* TLL1

**DOI:** 10.3390/ijms252312865

**Published:** 2024-11-29

**Authors:** Valiya Nadakkakath Agisha, Erinjery Jose Suraby, Savitha Dhandapani, Yee Hwui Sng, Shi Hui Lim, Bong Soo Park

**Affiliations:** Temasek Life Sciences Laboratory, National University of Singapore, Singapore 117604, Singapore; agisha@tll.org.sg (V.N.A.); ej.suraby@gmail.com (E.J.S.); savitha@tll.org.sg (S.D.); yeehwui@tll.org.sg (Y.H.S.); shihui@tll.org.sg (S.H.L.)

**Keywords:** *Penicillium olsonii* TLL1, phosphate deficiency, plant growth promotion, transcriptomics, proteomics

## Abstract

Beneficial fungi are promising tools for enhancing plant growth and crop yield in stressful environments. *Penicillium olsonii* TLL1 (POT1) was identified as a potential biofertilizer enhancing plant growth and phosphate use efficiency especially under phosphate deficiency stress. Hence, we attempted to explore bioinformatic insights into how POT1 enhances plant growth under phosphate starvation. In our study, wild-type *Arabidopsis thaliana* Columbia-0 roots and shoots cultivated with POT1 under phosphate-limiting conditions were employed for comparative analyses. By integrating transcriptomic and proteomic data, we identified key molecular pathways regulated by POT1 that influenced phosphate acquisition and plant stress tolerance. Comprehensive RNA-seq analysis revealed significant upregulation of genes involved in phosphate transport, root architecture, and stress-related pathways, while proteome profiling further highlighted proteins associated with lipid remodeling, phosphate metabolism, and phytohormone signaling. Bioinformatic analyses of differentially expressed genes (DEGs) and proteins (DEPs) elucidated the complex regulatory networks at both transcriptional and translational levels, with key contributions from auxin and ethylene signaling. Our study demonstrated that POT1-treated plants exhibited enhanced root development and nutrient uptake under phosphate-deficient conditions, driven by the coordinated regulation of phosphate solubilization genes and stress-responsive proteins. Our findings underscore the potential of multi-omics approaches in unraveling the molecular mechanisms behind plant–microbe interactions, with implications for improving sustainable agricultural practices.

## 1. Introduction

Plant-associated fungi of all lifestyles have evolved strategies including mechanisms of host communication, microbiome manipulation, and nutrient acquisition to colonize living or dead plant tissue and the surrounding substrate (i.e., soil). Hence, numerous biotechnological approaches have been developed to improve plant growth and health by offering safe, environmentally friendly alternatives, including those based on microorganisms. Implementing these strategies can significantly reduce the overuse of chemical fertilizers and pesticides. Despite the challenging environmental conditions, soil harbors a diverse array of microbial species from different domains of life. Although microbial diversity remains underestimated due to methodological limitations, microorganisms are crucial in both natural and cultivated soils, influencing soil quality and plant productivity [1,2]. However, long-term chemical fertilization and pesticide use have diminished soil microbial diversity and weakened beneficial microbe–plant interactions, which are an integral part of the plant holobiont [3]. As a result, restoring soil productivity by using bioeffectors, such as biostimulants or biofertilizers, has become a priority and is one of the most extensively researched biotechnological alternatives to traditional chemical fertilizers.

Biofertilizers, while defined in various ways, generally refer to plant-beneficial microorganisms and their metabolites, excluding biocontrol agents [4,5,6]. Among these, fungi are a highly diverse group of microorganisms that can adapt to various adverse environmental conditions, including salinity, drought, heavy metal contamination, and extreme pH levels [7,8,9]. In soil, an estimated 1.5 million fungal species exist, either as free-living organisms or endophytes within plant tissues [10]. Beneficial fungi, particularly those inhabiting the plant rhizosphere or endosphere, are promising tools for promoting plant growth in stressful environments [11,12,13]. Many of these plant growth-promoting fungi enhance plant health by improving plant physiology, development, and environmental adaptability [14,15]. They aid plants in various ways, such as producing and modulating phytohormones like indole-3-acetic acid (IAA) and gibberellins (GA); enhancing nutrient acquisition of elements like iron, phosphorus, and nitrogen; and producing 1-aminocyclopropane-1-carboxylic acid (ACC) deaminase [16,17,18]. For example, the endosymbiont *Piriformospora indica* has been reported to promote tomato growth by increasing levels of putrescine, IAA, and GA [19]. Additionally, beneficial fungi can indirectly enhance plant growth by suppressing phytopathogens and inducing systemic resistance. For instance, *Trichoderma harzianum* has been shown to reduce the severity of diseases caused by phytopathogenic fungi, bacteria and plant-parasitic nematodes, thereby contributing to the increased yield of several vegetable crops [20,21].

Phosphorus is a major macronutrient in plants, and inorganic phosphate (Pi) is the major available form of phosphorus in soil. Thus, the bioavailability of Pi affects plants’ performance, and they respond to phosphate limitation through the activation of morphological and physiological adaptations. In order to increase Pi availability, plant roots modify their root architecture by inhibiting primary root elongation and stimulating lateral roots and root hairs [22]. The activity of phytohormones including auxin and ethylene has been implicated in different aspects of these root developmental responses [23]. Furthermore, the systemic responses include enhanced Pi transport through the induction of high-affinity transporters and Pi mobilization through the activation of phosphatases and breakdown of phospholipids [24].

Among the plant-beneficial microbes, fungi are vital in aiding plant nutrient uptake including phosphorus uptake and improving plant health [1,25]. Many fungi can solubilize insoluble phosphates and facilitate phosphate acquisition by plants and, therefore, form an important part of commercial microbial products, with *Aspergillus*, *Penicillium*, and *Trichoderma* being the most efficient [26]. Our previous study focused on the role of phosphate-solubilizing microorganisms (PSMs) in promoting plant growth, particularly under phosphate-limiting conditions, and a newly identified fungal strain, *P*. *olsonii* TLL1 (POT1), which enhances plant growth and phosphate acquisition by solubilizing insoluble phosphates, was introduced [27]. When compared with other *Penicillium* strains like *P. bilaiae*, *P. chrysogenum*, *P. janthinellum*, and *P. simplicissimum*, POT1 had greater plant growth promotion and phosphate solubilizing activity. Moreover, we showed that it could be utilized as a high-potential biofertilizer for both monocot and dicot crop growth, as well as in a variety of soil types, including upland and paddy soil [27]. This effectiveness of POT1, particularly in phosphate-deficient soils, highlights its potential as a superior candidate for biofertilizer applications to support sustainable agriculture. Furthermore, POT1 mitigates aluminum toxicity and enhances plant growth under acidic soil conditions. We demonstrated that POT1 alleviated aluminum stress by detoxifying aluminum through organic acid production, significantly improving plant growth and nutrient uptake, thus offering potential applications in sustainable agriculture for acidic environments [13].

However, the molecular mechanism of the interactions between POT1 and plants under stress conditions is yet to be explored. Bioinformatics approaches will elucidate the molecular events and uncover the regulatory networks involved in the alleviation of stress responses by POT1. In this study, the potential role of the beneficial fungus POT1 in improving phosphate availability and promoting plant growth under phosphate-starved condition was deciphered through transcriptome and proteome analyses.

## 2. Results

### 2.1. Transcriptome Sequencing and Assembly

**Figure 1:**
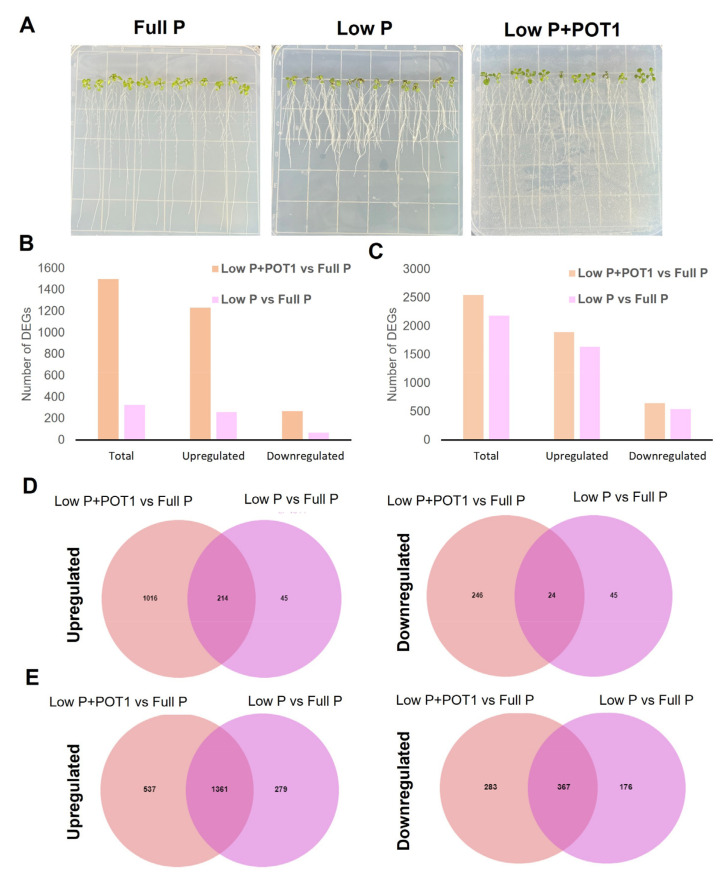
Transcriptome analysis of *Arabidopsis thaliana* upon *Penicillium olsonii* TLL1 (POT1) treatment. (**A**) *A. thaliana* grown under full P and low P conditions and with POT1 under low P conditions. Differentially expressed genes in (**B**) roots and (**C**) shoots of *A. thaliana* after inoculation with POT1. DEG analysis was performed by comparing the pairwise combinations low P + POT1 vs. full P and low P vs. full P. Venn diagrams were plotted to show the differentially co-expressed upregulated and downregulated DEGs in both (**D**) roots and (**E**) shoots.

*A. thaliana* cultivated on low P, full P, and low P + POT1 was used for transcriptome analysis (Figure 1A). A total of 1727.6 million (261 GB) raw reads of 151 bp read length were generated from 18 libraries (three biological replicates each from low P + POT1, low P, and full P). From root samples, 846.2 million reads (127.8 GB) were obtained, while shoot samples accounted for 881.4 million (133.2 GB) raw reads. Pre-processing of the raw data resulted in the removal of 1.45% of the total reads, and an average of 95% of the reads passed the ≥30 Phred score. An average of 95.9% and 98.3% of the trimmed reads of root and shoot samples, respectively, were mapped to the *A. thaliana* reference genome. Details of the raw data including GC%, Q20, and Q30 scores for each sample; trimmed reads; and mapping are given in Table S1. Assembly of the mapped reads then proceeded using StringTie based on the reference genome model, and gene/transcript expression levels in all samples were obtained. Out of 38,338 transcripts obtained through StringTie, a total of 20,338 high-quality transcripts were obtained for statistical analysis for differential gene expression profiling.

#### 2.1.1. Differential Gene Expression Profiling of *A. thaliana* in Response to POT1 Under P-Sufficient and P-Deficient Conditions

To identify the effects of POT1 on *A. thaliana* under P-deficient conditions, treated and untreated samples under P-deficient conditions were compared against untreated samples under P-sufficient conditions. Hence, DEG analysis was performed for two different pairwise comparisons, viz., low P + POT1 vs. full P and low P vs. full P, in both roots and shoots (Table S2). In roots, the number of total DEGs was abundant (4.57-fold) in POT1-treated samples as compared with the untreated samples under P-deficient conditions. We observed that both the upregulated genes (4.74-fold) and the downregulated genes (3.91-fold) were remarkably higher in response to POT1 (Figure 1B). In shoots, the number of total DEGs were slightly higher (1.17-fold) in response to POT1 compared with the untreated samples (Figure 1C). The top 50 upregulated and downregulated genes in the roots and shoots of low P + POT1 vs. full P and low P vs. full P are given in Table S3.

The differential expression of genes in *A. thaliana* in response to POT1 under P-deficiency stress was analyzed by performing the co-expression analysis between the pairwise combinations, low P + POT1 vs. full P and low P vs. full P. In roots, a larger number of unique DEGs were observed in response to POT1 (84.1%) compared with the untreated samples (27.4%), and a total of 238 DEGs were commonly expressed under both conditions. In shoots, 32.2% of the total DEGs under treated conditions were uniquely expressed, and a total of 1728 DEGs were common between treated and untreated samples. The results of co-expression analysis are summarized in Figure 1D,E and Table S4.

#### 2.1.2. GO and KEGG Enrichment of DEGs

**Figure 2:**
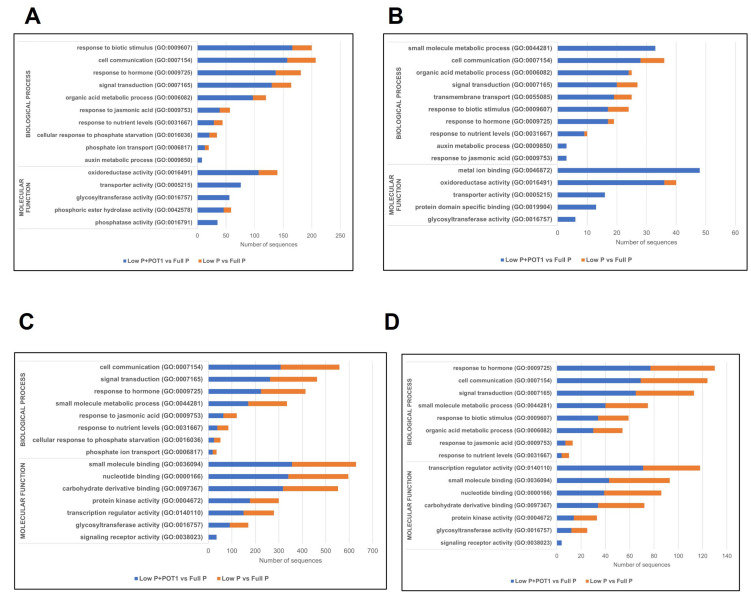
Gene ontology (GO) annotation of differentially expressed genes in *Arabidopsis thaliana* treated with *Penicillium olsonii* TLL1. GO categories in (**A**) upregulated and (**B**) downregulated DEGs in roots and (**C**) upregulated and (**D**) downregulated genes in shoots. The x-axis represents the number of sequences, and the y-axis represents the GO terms under the main categories, biological process and molecular function.

To understand the functions of the DEGs, GO enrichment analysis was performed in roots and shoots of *A. thaliana*, and GO categories enriched in different treatments were identified (Figure 2, Table S5). Although some of the GO terms, viz., oxidoreductase activity (GO:0016491) and phosphoric ester hydrolase activity (GO:0042578), under the molecular function (MF) category were enriched commonly in both low P + POT1 vs. full P and low P vs. full P, the number of sequences were comparatively higher in the roots of low P + POT1 vs. full P. The MF categories uniquely enriched in low P + POT1 vs. full P included phosphatase activity (GO:0016791), glycosyltransferase activity (GO:0016757), transporter activity (GO:0005215), etc. Similarly, the number of sequences under the GO terms, viz., response to biotic stimulus (GO:0009607), response to hormone (GO:0009725), signal transduction (GO:0007165), etc., under the biological process (BP) category were significantly higher in the roots of POT1-treated plants. The GO term, auxin metabolic process (GO:0009850), under the BP category was uniquely enriched in the roots of POT1-treated plants. In shoots, the GO terms under the MF category, viz., transcription regulator activity (GO:0140110), nucleotide binding (GO:0000166), etc., were substantially higher compared with the untreated plants, while the GO term signaling receptor activity (GO:0038023) was uniquely enriched. Among the GO terms under the BP category, signal transduction (GO:0007165) and response to hormones (GO:0009725) were prominent in the shoots of POT1-treated plants compared with the untreated plants.

**Figure 3:**
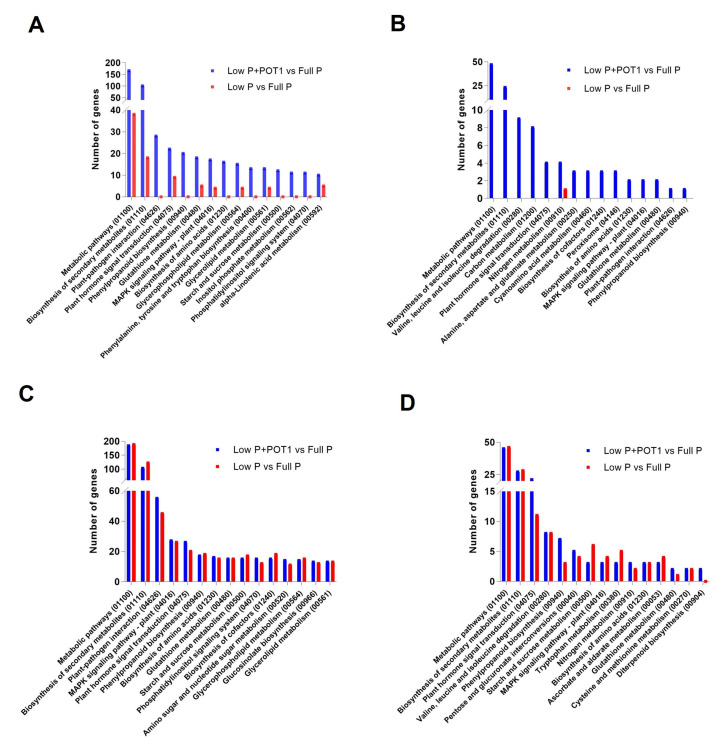
Kyoto Encyclopedia of Genes and Genomes (KEGG) pathway annotation of the differentially expressed genes in *Arabidopsis thaliana* treated with *Penicillium olsonii* TLL1. Distribution of KEGG pathways in (**A**) upregulated and (**B**) downregulated DEGs in roots and (**C**) upregulated and (**D**) downregulated DEGs in shoots. The x-axis represents the number of genes, and the y-axis represents the KEGG categories.

The KEGG analysis resulted in the identification of major pathways enriched in the POT1-treated plants (Figure 3, Table S5). The pathways, viz., metabolic pathways (01100) and biosynthesis of secondary metabolites (01110), were prominent in the POT1-treated roots compared with the untreated control. The pathways uniquely enriched in the roots of POT1-treated plants included phenylpropanoid biosynthesis (00940), biosynthesis of amino acids (01230), Inositol phosphate metabolism (00562), etc.

#### 2.1.3. Identification of Root Development-Related Genes Under Phosphate Starvation

**Figure 4:**
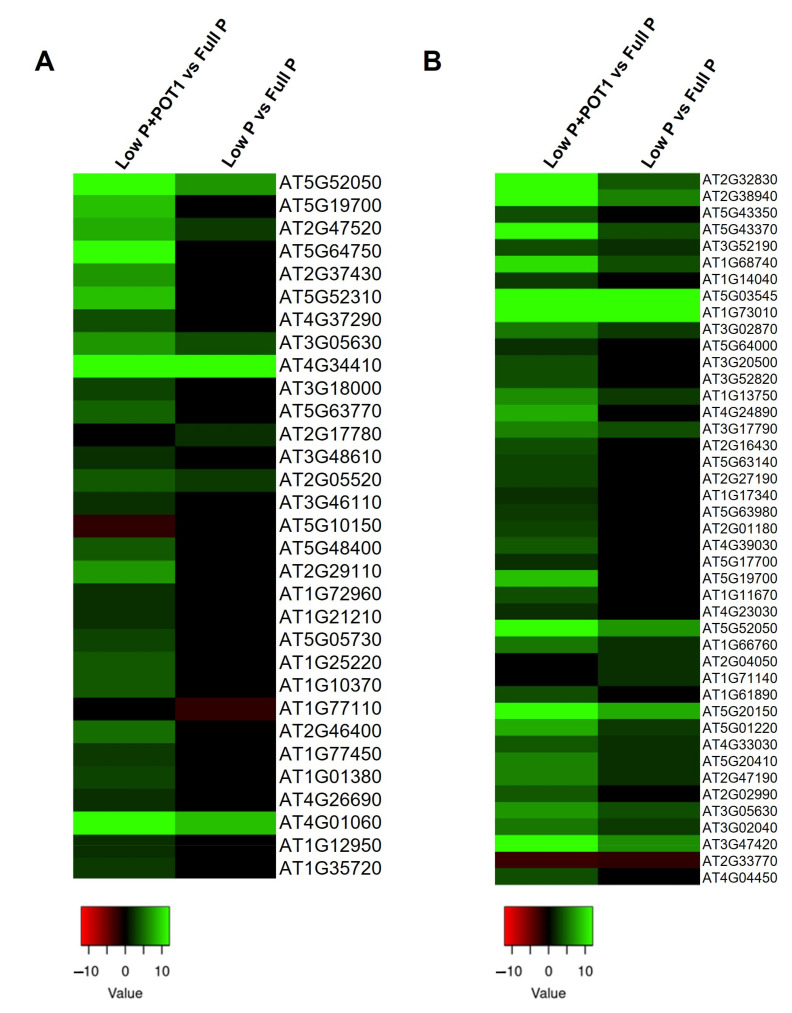
Heat map of the differentially expressed genes (DEGs) in *Arabidopsis thaliana* treated with *Penicillium olsonii* TLL1 (POT1) under low P conditions. (**A**) DEGs related to root development. (**B**) DEGs related to phosphate starvation in the pairwise comparisons, low P + POT1 vs. full P and low P vs. full P, in roots. The heat maps were constructed using the log2 fold change values, and the genes in green and red represent upregulated and downregulated genes, respectively.

We explored the genes involved in the modification of root system architecture especially in the roots of POT1-treated plants (Figure 4A, Table S6). Root growth development genes such as MATE efflux family protein, integrase-type DNA-binding superfamily proteins (*ERF71*, *ABR1*, and *RRTF1/ERF109*), and phospholipase D P2 (*PLDP2*) were more prominently overexpressed in the roots of POT1-treated plants. In addition, genes encoding glutamate receptor family proteins were specifically upregulated in POT1-inoculated plants. Interestingly, the genes involved in lateral root formation such as anthranilate synthase alpha subunit 1 (*ASA1*) and WRKY DNA-binding protein 46 (*WRKY46*) were exclusively induced in POT1-treated plants. Moreover, genes that increased the root hair length and density such as homeodomain-like superfamily protein (*ETC1*) and root hair specific 2 (*RHS2*) were specifically expressed, while CAPRICE-like MYB3 (*CPL3*) was more prominently upregulated upon POT1 treatment.

#### 2.1.4. Comparative Expression Analysis of Phosphate Starvation-Related Genes

In our study, several genes related to phosphate starvation, viz., phosphate transporters and phosphatase genes, were regulated in response to POT1 (Figure 4B and Figure S1A, Table S6). Genes involved in phosphate transport such as phosphate transporter genes (*PHT1-1*, *PHT1-2*, *PHT1-4*, and *PHT1-5*), phosphate transporter traffic facilitator 1 (*PHF1*), EXS (ERD1/XPR1/SYG1) family protein (*PHO1*), etc., showed much higher expression in the roots of POT1-treated plants compared with the untreated plants. The expressions of all these genes were comparatively lower in the shoots of POT1-treated plants. Interestingly, the phosphate 2 gene (*PHO2*) involved in the degradation of *PHO1* was downregulated, and its expression level was slightly lower in the roots of POT1-treated plants compared with the untreated plants. In shoots, *PHO2* was induced in both treated and untreated plants, and it did not show a significant difference between them. Importantly, another gene expressed in response to phosphate starvation protein (*ATIPS2*) was consistently upregulated and showed greater expression in both the roots and shoots of POT1-inoculated plants. The expression of the transcription factors, viz., WRKY family transcription factor (*WRKY42*) and myb domain protein 2 (*MYB2*), regulating phosphate starvation responses was also examined. *MYB2* was found to be enhanced in both roots and shoots, whereas *WRKY42* was induced only in the roots in response to POT1. Among the phosphatase genes, inorganic pyrophosphatase 1 (*PS2*), which catalyzes the recycling of pyrophosphate (PPi), showed higher expression in the roots of the treated compared with the untreated plants, while consistently high expression was noticed in the shoots of both the treated and untreated plants. Other induced phosphatase genes in the roots upon POT1 treatment included inositol monophosphatase family protein (*VTC4*), SAL1 phosphatase-like protein, purple acid phosphatase (PAP) genes (*PAP10*, *PAP12*, *PAP17*, *PAP18*, *PAP22*, *PAP24*, and *PAP29*), phosphatidic acid phosphatase 1 (*PAP1*), etc. Remarkably, the expressions of inositol polyphosphate 5-phosphatase 11 (*5PTASE11*) and myo-inositol polyphosphate 5-phosphatase 2 (*IP5PII*) were comparatively higher only in the shoots upon POT1 treatment. Moreover, the expression of several phosphate starvation-related proteins such as SPX domain-containing protein 1 (*SPX1*), ribonuclease 1 (*RNS1*), and putative glycerol-3-phosphate transporter 1 (*G3Pp1*) were comparatively higher in the roots of POT1-treated plants, while they showed decreased expression in the shoots upon POT1 treatment. Similarly, genes involved in lipid remodeling such as sulfoquinovosyldiacylglycerol synthase (*SQD1* and *SQD2*) and monogalactosyldiacylglycerol synthase 2 (*MGD2*) also showed significantly higher upregulation in the roots upon POT1 treatment, whereas their expression levels were slightly lower in the shoots compared with the untreated plants.

#### 2.1.5. Differential Expression Profiling of Phytohormone-Related Genes

**Figure 5:**
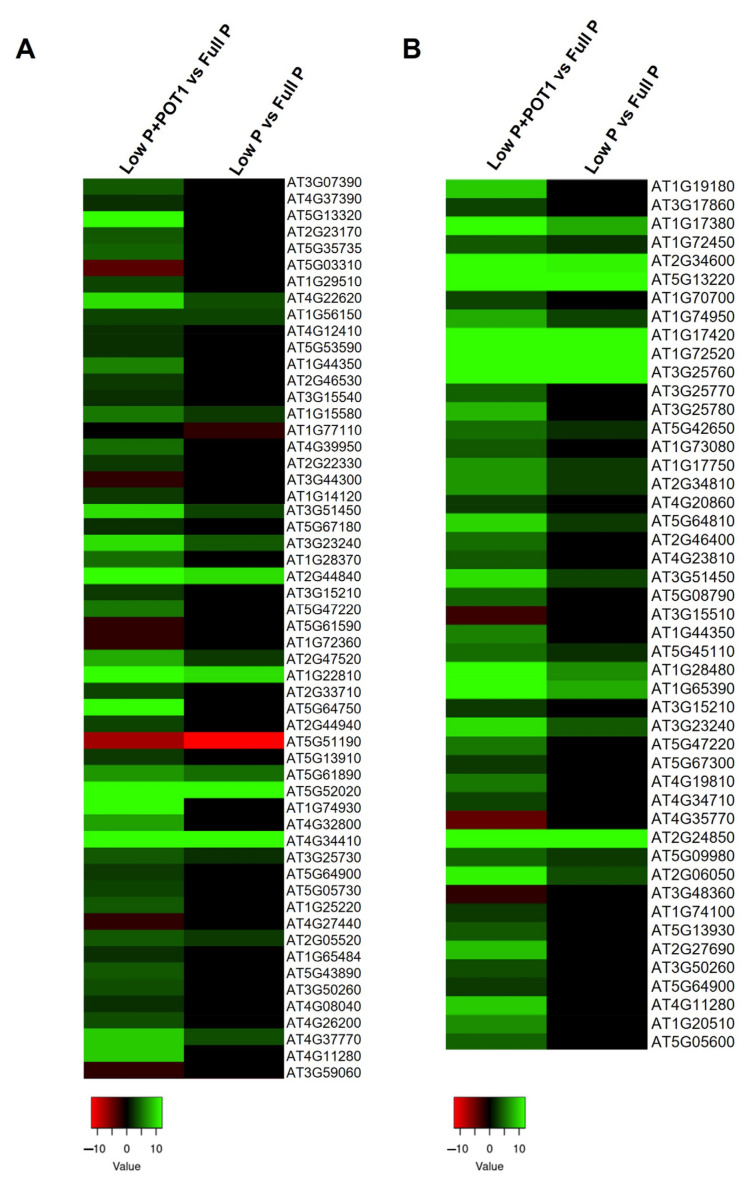
Heat map of the differentially expressed genes (DEGs) in *Arabidopsis thaliana* treated with *Penicillium olsonii* TLL1 (POT1) under low P conditions. (**A**) DEGs related to auxin and ethylene phytohormones. (**B**) DEGs related to jasmonic acid in the pairwise comparisons, low P + POT1 vs. full P and low P vs. full P, in roots. The heat maps were constructed using the log2 fold change values, and the genes in green and red represent upregulated and downregulated genes, respectively.

Interestingly, several genes associated with auxin and ethylene were differentially regulated in both roots and shoots in response to POT1 treatment (Figure 5A and Figure S1B, Table S6). Auxin-related genes, viz., auxin-responsive GH3 family proteins, IAA-amino acid hydrolase ILR1-like 6 (*ILL6*), auxin-induced in root cultures-like protein (*AIR12*), and cytochrome P450 (*CYP79B2* and *CYP79B3*), were greatly upregulated in both the roots and shoots by POT1. Differential regulation of genes encoding some of the SAUR-like auxin-responsive family proteins were also observed in the roots and shoots following POT1 treatment. POT1 modulated the expression of several genes involved in the ethylene biosynthetic process and ethylene signaling pathway. For instance, 1-aminocyclopropane-1-carboxylate synthase genes, viz., *ACS6*, *ACS7*, *ACS8*, and *ACS11*, were induced in the roots and *ACS2*, *ACS6*, and *ACS7* were induced in the shoots of POT1-treated plants. Moreover, several members of the ethylene-responsive transcription factors (*ERF*) including *ABR1*, *RRTF1/ERF109*, and *ERF114* were significantly enhanced in both the roots and shoots upon POT1 treatment, while *ERF71* and *LEP* were activated only in the roots.

Furthermore, the expression level of gibberellin (GA)-related genes was observed to be regulated by POT1 (Table S6). Among the genes involved in the catabolism of GA, *GA2OX6* was enhanced in both the roots and shoots, while *GA2OX4* and *GA2OX2* were enhanced in the roots and shoots, respectively. Genes involved in the production of bioactive GAs, namely, *GA20OX2*, were downregulated in both the roots and shoots, while *GA3OX1* was downregulated only in the roots. In the shoots, genes encoding DELLA proteins, which act as repressors of the GA signaling pathway, viz., *RGL1* and *RGL3*, were induced more prominently by POT1.

We observed that the expression of genes involved in JA biosynthesis, viz., allene oxide synthase (*AOS*), allene oxide cyclase (*AOC1*, *AOC2*, and *AOC3*), and lipoxygenase (*LOX3* and *LOX4*), was upregulated under Pi starvation conditions. The expression of these genes was further activated in the roots and shoots following POT1 treatment (Figure 5B and Figure S1C, Table S6). Interestingly, genes encoding jasmonate-zim-domain proteins involved in JA signaling, viz., *JAZ1*, *JAZ2*, *JAZ3*, *JAZ5*, *JAZ6*, *JAZ7*, *JAZ9*, and *JAZ10*, were also consistently activated in both the roots and shoots by POT1. In addition, PEP1 receptors (*PEPR1* and *PEPR2*) induced in response to JA were enhanced in both the roots and shoots by POT1 inoculation. Some of the transcription factors such as *WRKY51* and *WRKY53* were more activated in both the roots and shoots of the POT1-treated plants, while *WRKY11* was enhanced only in the shoots.

#### 2.1.6. qPCR Validation of RNA-seq Data

**Figure 6:**
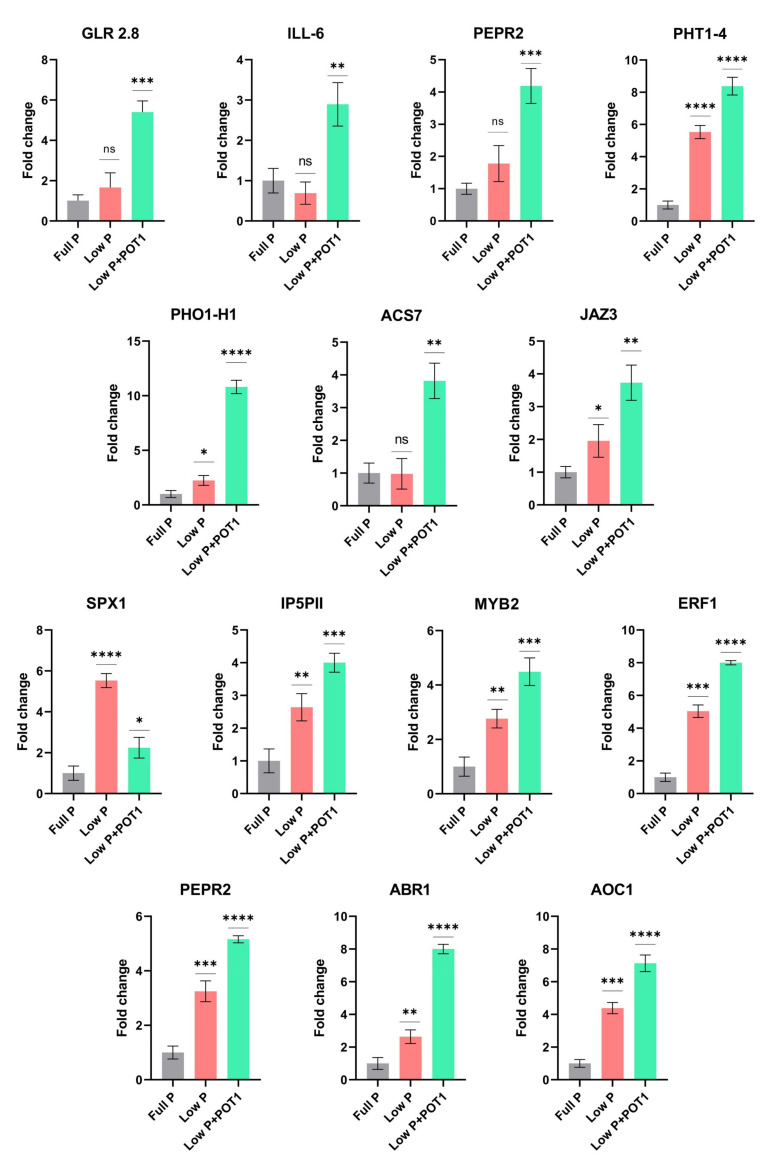
Validation of differentially expressed genes identified in Illumina sequencing by qPCR. Genes selected for validation in *Arabidopsis thaliana* roots include glutamate receptor 2.8 (*GLR2.8*), IAA-amino acid hydrolase ILR1-like 6 (*ILL-6*), PEP1 receptor 2 (*PEPR2*), phosphate transporter 1;4 (*PHT1-4*), phosphate transporter PHO1 homolog 1 (*PHO-H1*), 1-amino-cyclopropane-1-carboxylate synthase 7 (*ACS7*), and jasmonate-zim-domain protein 3 (*JAZ3*). In shoots, the expressions of SPX domain-containing protein 1 (*SPX1*), myo-inositol polyphosphate 5-phosphatase 2 (*IP5PII*), MYB domain protein 2 (*MYB2*), ethylene response factor 1 (*ERF1*), *PEPR2*, ethylene-responsive transcription factor ABR1 (*ABR1*), and allene oxide cyclase 1 (*AOC1*) were validated. The x-axis represents the treatments, and the y-axis represents the fold change calculated by qPCR using the actin gene as the reference gene. A Student’s *t*-test was used to assess the data in replicates, and an unpaired two-tailed *t*-test was used to determine significant differences across treatments (*n* = 3; **** *p* < 0.0001; *** *p* < 0.001; ** *p* < 0.01; * *p* < 0.05); ns, Non-Significant.

To further validate the RNA-seq results, we selected seven differentially expressed genes (DEGs) from both roots and shoots for qPCR analysis. As illustrated in Figure 6, the qPCR results showed a similar expression trend to the RNA-seq results (Table S7), confirming the accuracy of the Illumina sequencing analysis. Specifically, the DEGs *GLR2.8*, *ILL6*, *PEPR2*, *PHT1-4*, *PHO1-H1*, *ACS7*, and *JAZ3* were markedly enhanced in the roots following POT1 treatment. In the shoots, the expression of genes annotated as *SPX1*, *IP5PII*, *MYB2*, *ERF1*, *PEPR2*, *ABR1*, and *AOC1* were validated by qPCR.

### 2.2. Proteome Profiling of POT1-Treated A. thaliana

**Figure 7:**
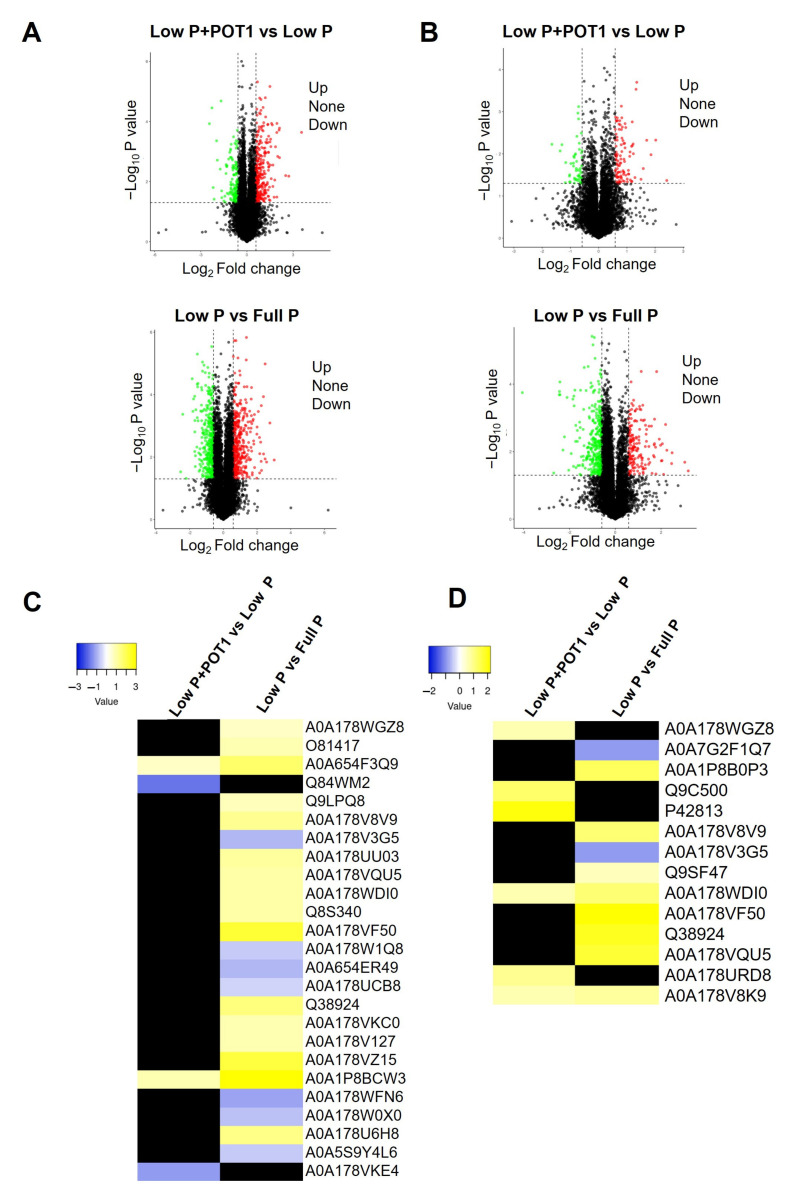
Proteome analysis of *Arabidopsis thaliana* treated with *Penicillium olsonii* TLL1 (POT1). Volcano plots representing the differentially expressed proteins in the pairwise combinations, low P + POT1 vs. low P and low P vs. full P, in (**A**) roots and (**B**) shoots are shown. Proteins exhibiting a fold change greater than 1.5 and less than 0.67 with a *p* value of <0.05 were considered significantly differentially expressed for upregulated and downregulated proteins, respectively. Heat maps of the differentially expressed proteins (DEPs) related to plant growth in the (**C**) roots and (**D**) shoots of *A. thaliana* treated with POT1 under low P condition are represented. The heat maps were constructed using the log2 fold change values, and the genes in yellow and blue represent upregulated and downregulated genes, respectively.

We performed proteome analysis to better understand the molecular responses in *A. thaliana* in response to POT1, and a total of 11,753 proteins were identified. Differential expression analysis was performed for two pairwise combinations in both roots and shoots, low P + POT1 vs. low P and low P vs. full P (Table S8). Overall, 494 proteins showed differential expression in roots of fungus-treated plants compared with untreated plants under phosphate stress, among which 321 were upregulated and 173 were downregulated, while in shoots, 213 proteins were differentially expressed, among which 140 were upregulated and 73 were downregulated. Likewise, in low P vs. full P, a total of 821 proteins (406 upregulated and 415 downregulated) and 603 proteins (205 upregulated and 398 downregulated) were differentially expressed in roots and shoots, respectively. Volcano plots representing the DEPs are given in Figure 7A,B.

GO enrichment analysis was performed for DEPs in roots and shoots of *A. thaliana*, and GO categories enriched in different treatments were identified (Figures S2–S5, Table S9). In roots, the GO terms, viz., protein phosphorylation, transmembrane transport, protein kinase activity, and oxidoreductase activity, were among the highly enriched GO terms in low P + POT1 vs. low P, while in shoots, cysteine-type peptidase activity under molecular function was the highly enriched GO term. In low P vs. full P, the GO terms, RNA binding, transferase activity, and cellular carbohydrate metabolic process were predominantly enriched in roots. On the other hand, the metabolic process under the biological process category was enriched in shoots. The KEGG analysis resulted in the identification of major pathways enriched in the DEPs of POT1-treated plants (Figures S6–S9, Table S9). In the low P + POT1 vs. low P comparison, plant–pathogen interaction, purine metabolism, and glyoxylate and dicarboxylate metabolism were among the overrepresented pathways in roots. In shoots, pathways such as plant hormone signal transduction, amino sugar and nucleotide sugar metabolism, and protein processing in the endoplasmic reticulum were overrepresented in low P + POT1 vs. low P. In the low P vs. full P comparison, metabolic pathways and biosynthesis of secondary metabolites were abundantly enriched in both roots and shoots.

Functional annotation clustering of the top 50 differentially expressed proteins (DEPs) (Table S10) identified clustering groups with medium classification stringency. Several proteins in the low P + POT1 vs. low P comparison were associated with transferase activity, protein kinase activity, and transmembrane functions in both roots and shoots (Figures S10 and S11). In contrast, in the low P vs. full P comparison, hydrolase activity, transmembrane function, and signaling were commonly represented in both roots and shoots (Figures S12 and S13).

We explored the proteins involved in plant growth and phosphate starvation in POT1-treated plants (Figure 7C,D, Table S11). Proteome profiling revealed comparatively fewer proteins, in contrast to the RNA-seq data. Notably, non-specific phospholipase C4 (NPC4) and SQD2 were upregulated in the roots. On the other hand, shoots showed increased expression of phosphate starvation-related proteins, such as RNS1 and purple acid phosphatase. Additionally, phytohormone-related proteins, including auxin response factor and allene oxide cyclase, were also enhanced.

### 2.3. Nutrient Profiling in POT1-Treated Plants

**Figure 8:**
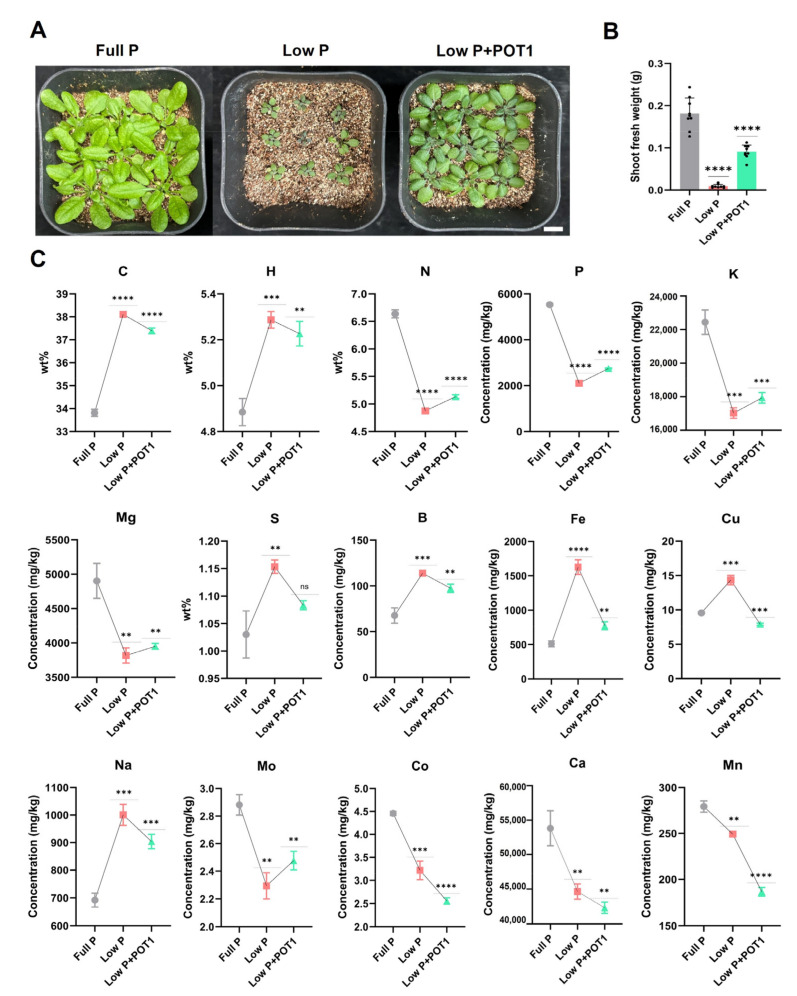
Nutrient profiling in *Arabidopsis thaliana* with *Penicillium olsonii* TLL1 (POT1) treatment. (**A**) *A. thaliana* grown in vermiculite for 2 weeks under full P and low P conditions and with POT1 under low P conditions. The scale bar represents 1 cm, and the bar diagram shows their respective (**B**) shoot fresh weight (*n* = 9; unpaired two-tailed *t*-test, **** *p* < 0.0001). (**C**) Determination of nutrients in the shoots of *A. thaliana* treated with POT1 under low P conditions. *A. thaliana* plants were grown for 2 weeks in vermiculite treated with POT1, and nutrient profiling was performed from three replicates (*n* = 3; unpaired two-tailed *t*-test, **** *p* < 0.0001; *** *p* < 0.001; ** *p* < 0.01); ns, Non-Significant.

We were able to identify the changes in shoot nutrient elements under distinct phosphate conditions (Figure 8). Elemental analysis showed that concentrations of N, P, and K were increased in low P shoots upon POT1 treatment, although they were significantly lower in untreated low P than in full P shoots. On the other hand, the concentrations of C, S, B, Na, Cu, and Fe were lowered in the shoots of POT1-treated plants under low P even though they were substantially higher in untreated low P than full P. Furthermore, POT1 treatment decreased the amounts of Co, Ca, and Mn that had already decreased under low phosphate deficiency stress.

## 3. Discussion

### 3.1. POT1 Regulates the Expression of Root Development-Related Genes Under Phosphate Starvation

In our study, several genes involved in root development were regulated by POT1 correlating with the root elongation under Pi starvation conditions (Figure 4A). Genes encoding MATE efflux family proteins are expressed to overcome phosphate starvation through citrate exudation and impact the root system architecture [28]. The secretion of citric acid is one of the mechanisms for improved phosphate acquisition from insoluble phosphates. Hence, the enhanced expression of *MATE* genes by POT1 suggested that POT1 promoted the synthesis and expression of citrate to cope with low Pi. The genes encoding *ERF71*, *ABR1*, and *RRTF1 (ERF109)* induced by POT1 belong to the integrase-type DNA-binding superfamily of proteins and are associated with root development. The gene *ERF71* was reported to play an important role in root development through root cell expansion [29]. Other ERFs such as *ABR1* and *RRTF1/ERF109* were identified to promote auxin production and root regeneration by activating *ASA1* [30]. The *PLDP2* gene contributed to the supply of inorganic phosphorus for cell metabolism, and diacylglycerol moieties for galactolipid synthesis in phosphorus-starved roots were reported to be involved in root elongation during phosphate limitation [31]. Our study demonstrated that POT1 significantly enhanced phosphate uptake efficiency and stress response mechanisms in plants under phosphate starvation. Notably, genes involved in root development, such as *MATE* efflux and *PLDP2*, contributed to improved root architecture and phosphate acquisition. Genes involved in lateral root formation, viz., *ASA1* and *WRKY46*, were also induced by POT1 under phosphate-deprived conditions. The *ASA1* was essential for jasmonate-induced auxin biosynthesis during lateral root development [32], while transcription factor *WRKY46* was reported to modulate the development of *A. thaliana* lateral roots in osmotic/salt stress conditions via regulation of ABA signaling and auxin homeostasis [33], suggesting the involvement of auxin in root development by POT1. Additionally, some of the genes involved in root hair formation were greatly enhanced by POT1. For instance, genes such as *ETC1*, *CPL3*, and *RHS2* were associated with the overproduction of root hairs resulting in the exploration of more soil surface and the acquisition of available Pi [34,35]. The induction of these root hair-enhancing genes with POT1 indicated its strategy to enhance soil penetration for better acquisition of available Pi.

### 3.2. POT1 Maintains the Pi Homeostasis by Modulating the Phosphate Starvation-Related Genes

The comparative expression analysis of phosphate starvation-related genes revealed the role of genes involved in Pi homeostasis during POT1 plant growth promotion (Figure 4B). The phosphate transporter genes, viz., *PHT1-1* and *PHT1-4*, which play major roles in the uptake of inorganic phosphate under phosphate limiting conditions [36], *PHF1* facilitating the trafficking of *PHT1-1* from the endoplasmic reticulum to the plasma membrane [37], and *PHO1* contributing to the transport of inorganic phosphate (Pi) into the root xylem vessels [38], were highly induced in the roots of POT1-treated plants, indicating the increased translocation and acquisition of phosphate by POT1 at the site of colonization. PHO2 is a ubiquitin-conjugating enzyme E2 that mediates PHO1 degradation in response to Pi availability [39,40]. Hence, the downregulation of *PHO2* was correlated with the induction of *PHO1*, indicating the enhanced Pi uptake to maintain Pi homeostasis under low P conditions in the roots of POT1-treated plants. Furthermore, *WRKY42*, which controls *PHO1* and *PHT1-1* expression [41], was induced exclusively in POT1-treated plants, indicating increased root Pi uptake by POT1 under low P conditions. Another transcription factor MYB2, which activates the expression of the microRNA*399* gene that represses *PHO2* [42], was induced and can be corroborated with the lower levels of *PHO2* in the roots of POT1-treated plants compared with the untreated plants. The induced expression of *ATIPS2* in the roots and the shoots of POT1-treated plants strongly indicated increased Pi uptake and phosphate distribution between roots and shoots. The induction of *PS2*, *VTC4*, and *SAL1* phosphatase-like protein, which are involved in Pi-recycling, suggested that it might be an important strategy for plant adaptation by POT1 under Pi-deficiency stress. To cope with Pi deficiency, plants produce purple acid phosphatases, which enhance internal Pi remobilization or the release of Pi from external organophosphates [43]. Therefore, the overexpression of *PAP* genes encoding the purple acid phosphatases suggested the increased phosphate homeostasis in the roots by POT1. In shoots, phosphatases, viz., *5PTASE11* and *IP5PII*, which hydrolyzes 5-phosphates from inositol phosphates and phosphoinositide substrates, were induced confirming the increased phosphate acquisition by POT1. Furthermore, *SPX1*, which acts as an intracellular Pi sensor by inhibiting PHR1 DNA-binding activity [44], was induced in the roots, indicating the role of Pi sensing in phosphate acquisition by POT1 under phosphate-limiting conditions. The *RNS1* gene, which plays a crucial role in phosphate remobilization under Pi-limited conditions [45], was also induced in the POT1-treated roots. In addition, the *G3Pp1* appeared to be involved in the transport of organic P under Pi-deprived conditions [46] and was induced specifically in the roots by POT1. Under low P conditions, lipid remodeling is triggered, resulting in the release of phosphorus-free sulfoquinovosyldiacylglycerol (SQDG) and monogalactosyldiacylglycerol (MGDG) in the plastids, and the genes involved in this glycerolipid homeostasis such as *SQD1*, *SQD2*, and *MGD2* are thus considered as markers of low Pi response [47]. Here, the activity of these enzymes was enhanced in the roots of POT1-treated plants, suggesting their involvement in improving the internal Pi content in Pi-starved plants.

While these responses enhance Pi uptake, they impose significant metabolic costs. The activation of high-affinity phosphate transporters (*PHT1* family) and phosphate recycling genes, such as *ATIPS2*, *PS2*, *VTC4*, and *SAL1*, requires substantial ATP investment, which could otherwise support growth and stress responses. These processes, particularly lipid remodeling, may limit biomass production and secondary metabolism under sustained Pi stress. Additionally, the increased demand for Pi may interfere with the uptake of other essential nutrients, leading to potential imbalances. Despite these beneficial adaptations for low-P survival, the metabolic costs and trade-offs, including nutrient imbalances, require further investigation for optimizing POT1 inoculation in sustainable agriculture.

### 3.3. Phytohormone-Related Genes Play a Crucial Role in the Plant Growth Promotion by POT1

Auxin is important for many aspects of plant development and is required for altering primary root growth and in promoting root hair and lateral root formation. The induction of several auxin-related genes encoding auxin-responsive GH3 family protein, ILL6, SAUR-like auxin responsive proteins, etc., by POT1 (Figure 5A) suggested the involvement of auxin in plant growth promotion by POT1. The auxin-responsive GH3 family proteins might be engaged in catalyzing the production of indole-3-acetic acid (IAA)–amino acid conjugates, offering an approach for the plant to cope with the excess auxin produced following POT1 inoculation. On the other hand, ILL6 has been shown to hydrolyze JA– and IAA–amino acid conjugates and is involved in the turnover of JA and IAA, probably resulting in the accumulation of biologically active IAA and JA. Additionally, SAUR-like auxin-responsive proteins were also regulated in the POT1-treated plants, confirming the auxin-induced cell expansion leading to plant growth promotion by POT1. Another auxin-related gene induced by POT1, *AIR12*, was reported to have accumulated during auxin-induced lateral root formation in *A. thaliana* [48], suggesting the role of auxin in lateral growth by POT1. Moreover, significant upregulation of cytochrome P450 (*CYP79B2* and *CYP79B3*) involved in the biosynthesis of IAA also strongly suggested the induction of auxins following POT1 inoculation. Overall, it was evidenced that the auxin played a vital role in plant growth promotion by POT1 under phosphate starvation conditions.

It is a proven fact that along with auxin, ethylene is also involved in altering plant growth. Particularly, ethylene is required for cell expansion and for maintaining root growth under phosphate deficiency [49]. Here, the induction of ACS enzymes catalyzing the synthesis of ethylene clearly denoted the role of ethylene in growth promotion in *A. thaliana* by POT1. Additionally, the genes involved in the ethylene signaling pathway, viz., *ERF71*, *ABR1*, *RRTF1 (ERF109)*, *LEP*, and *ERF114* (Figure 5A), were induced by POT1. Among these, ERFs, *ERF71*, *ABR1*, and *RRTF1 (ERF109)* were reported to play important roles in root development [29,30]. The *LEP* transcription factor contributes to root development by promoting the number of xylem vessels [50], and *ERF114* affects cell proliferation via the activation of genes involved in cell cycle regulation and dormancy breaking [51]. Altogether, the enhanced expression of all these genes denoted the regulation of ethylene signal transduction pathways involved in plant growth by POT1.

Gibberellins (GAs) play a central role in plant growth and regulate a wide range of plant developmental processes. Recent studies demonstrated that GA participates in plant responses to stress conditions, and low-P stress in *A. thaliana* was found to be associated with the induction of GA deactivation genes and a decline in the expression of GA biosynthesis genes [52]. In this study, POT1 was found to regulate the endogenous level of gibberellins under P-limiting conditions. Upregulation of *GA2OX6*, *GA2OX4*, and *GA2OX2* catalyzing the 2-beta-hydroxylation of several biologically active gibberellins and downregulation of *GA3OX1* and *GA20OX2* involved in the production of bioactive GA indicated the decrease in gibberellin production upon POT1 treatment. In line with this, it has been reported that ectopic expression of nuclear factor Y subunit alpha 8 (NF-YA8) in *A. thaliana* was associated with the downregulation of genes involved in the production of active GAs and upregulation of genes involved in the conversion of active GAs to inactive GAs resulting in increased lateral root emergence under low P [53]. Hence, the decrease in the active GAs might be related to an increase in lateral roots and root density following POT1 treatment. In addition, POT1 also led to the induction of DELLA proteins (RGL1 and RGL3) that act as repressors of the GA signaling pathway in shoots.

### 3.4. POT1 Activates the Jasmonic Acid-Related Genes in A. thaliana Under Phosphate Starvation Conditions

The expression pattern of genes involved in jasmonic acid biosynthesis and signaling during POT1 treatment (Figure 5B) suggested their potential role under Pi-deficient conditions. Although the majority of these genes were induced under P-deficient conditions compared with P-sufficient conditions, a significant increase in the expression was noticed upon POT1 treatment. This could be correlated to the induction of JA-related genes, viz., *AOS*, *AOC1*, *AOC1*, *LOX2*, *LOX4*, *JAZ*1, and *JAZ10* genes, in Pi-starved *A. thaliana* plants and the activation of these genes following Pi resupply [47]. Differential expression of genes in the JA pathway under Pi deficiency has been found to be associated with increased resistance to insect herbivory [54], suggesting the induction of defense responses by POT1. Moreover, *PEPR1* and *PEPR2*, which act as receptors for PEP defense peptides, were consistently induced by POT1 in both roots and shoots. These are involved in PAMP-triggered immunity (PTI) signaling and contribute to defense responses in *A. thaliana* [55], thus confirming the enhancement of defense responses in *A. thaliana* upon POT1 treatment.

### 3.5. Modulation of Proteome by POT1 Under Low-Phosphate Stress

Although there was a low correlation between proteomic and transcriptomic results, a few overlapping proteins were identified at the translational level through proteome analysis. The upregulation of SQD2, a marker of low-phosphate (Pi) response [47], was consistent with the RNA-seq results, confirming the involvement of lipid remodeling in enhancing Pi content in POT1-treated plants. Another upregulated protein in the roots was non-specific phospholipase C4 (NPC4), a phospholipid-hydrolyzing enzyme. It has been reported to promote primary root growth and root hair elongation in Pi-deprived *A. thaliana* [56]. The enhanced expression of this gene in both the RNA-seq and proteome profiles reaffirmed the role of phospholipid breakdown in providing Pi for essential functions during POT1 colonization under Pi deprivation. Purple acid phosphatases and RNS1 [43,45] have been associated with Pi remobilization under Pi-limited conditions. Proteome analysis of the shoots revealed the involvement of these proteins in Pi homeostasis during POT1-mediated plant growth under Pi-limited conditions. Moreover, the enhanced expression of auxin response factors and allene oxide cyclase confirmed the participation of the auxin signaling pathway and jasmonic acid biosynthetic process in POT1-mediated responses, as observed in the transcriptome analysis. However, as is consistent with many previous studies, the overlap between the transcriptome and proteome data is quite low, suggesting that post-transcriptional and post-translational regulatory processes play a key role in governing the complexity of protein synthesis. The low correlation between transcriptome and proteome data might also be attributed to technical issues affecting protein recognition and quantitative analysis, as well as biological variables [57]. For this reason, comprehensive studies involving metabolomic and immunological analyses will be helpful for further confirmation.

### 3.6. Effect of POT1 on Nutrient Acquisition Under Pi-Deprived Conditions

Elemental profiling provided a better understanding of the modulation of nutrients by POT1 under low P conditions. Macronutrients such as nitrogen (N), phosphorus (P), and potassium (K), which were reduced under phosphate deficiency stress, showed a notable increase following POT1 treatment, indicating improved nutrient acquisition. As demonstrated earlier by Suraby et al., the increase in P content was attributed to the phosphate-solubilizing capability of POT1. Additionally, the stimulated root development by POT1 might have led to better absorption of N and K from the soil. Similarly, the plant growth-promoting fungus *Penicillium pinophilum* was found to increase N, P, and K uptake, resulting in improved growth in pomegranate [58]. Phosphate deficiency in *A. thaliana* has been shown to increase iron (Fe) content, affecting primary root growth in response to Pi deficiency [59]. In our study, along with Fe, additional elements elevated during Pi deficiency—such as copper (Cu), carbon (C), sulfur (S), boron (B), and sodium (Na)—significantly decreased following POT1 treatment, implying that the stress responses were partially alleviated. Interestingly, cobalt (Co), calcium (Ca), and manganese (Mn) concentrations were highest in full P plants and lowest in low P + POT1-treated plants. Micronutrients like Mn and Co often share common transport systems, such as the NRAMP (Natural Resistance-Associated Macrophage Protein) family or high-affinity phosphate–metal co-transporters, which can influence their uptake dynamics. POT1 could modulate these transport systems, altering competitive uptake among these metals and potentially prioritizing phosphate over these micronutrients [60,61]. Similarly, calcium availability may also be affected by altered rhizosphere chemistry, potentially due to competition with solubilized phosphate for root transport sites.

## 4. Materials and Methods

### 4.1. Plant and Fungal Material and Growth Conditions

*P. olsonii* POT1 isolated from indoor dust samples [27] was cultured on Malt Extract Agar (MEA; pH—5.6) at 28 ± 2 °C for 7 days. For seedling growth, wild-type *A. thaliana* Columbia (Col-0) seeds obtained from the Arabidopsis Biological Resource Center (ABRC), Ohio State University (https://abrc.osu.edu/ (accessed on 11 November 2020)), were germinated on media containing Murashige and Skoog salts, 0.25 mM MES, 10 g/L sucrose, and 0.8% agar and grown at 22 °C under the photoperiod of 16 h light/8 h dark [40].

For plant growth, agar medium with modified Hoagland’s solution under different phosphate conditions, viz., low P (P-deficient condition, pH 5.6) containing 0.01 mM of KH_2_PO_4_ (Sigma-Aldrich, St. Louis, MI, USA) and full P (P-sufficient condition, pH 5.6) containing 0.50 mM of KH_2_PO_4_, was used [62]. For fungal treatment, low P agar medium was inoculated by spreading with 100 μL of POT1 spore suspension (10^8^ spores/mL) and incubated at 28 ± 2 °C. After 7 days, four-day-old seedlings were transferred onto the medium and grown for 10 days at 22 °C. Concurrently, plants grown on low P agar medium without fungal treatment were also maintained. Plants grown on untreated full P medium served as the control. Root and shoot samples comprising three biological replicates were harvested from both treated and untreated plants and used for proteome and transcriptome analyses.

### 4.2. RNA-Seq Profiling

The total RNA from the root and shoot samples was extracted using a FavorPrep^TM^ plant total RNA minikit (Favorgen, Ping-Tung, Taiwan). A total of 18 cDNA libraries corresponding to the treated and control (untreated) plants were constructed using a TruSeq Stranded mRNA LT Sample Prep Kit (Illumina, San Diego, CA, USA). The cDNA libraries were sequenced using Novaseq 6000 platform (Illumina, San Diego, CA, USA) to generate paired-end reads (2 × 151 bp) of about 12–15 GB quality reads/library. The quality of the raw reads obtained was analyzed using FastQC v0.11.7, and the raw reads were pre-processed by trimming the low-quality bases and adapter sequences using Trimmomatic 0.38. Using the sliding window method, bases of reads that did not qualify for window size 4 and mean quality 15 were trimmed. Reads with lengths shorter than 36 bp were dropped to produce trimmed data. The trimmed reads were mapped to the *A. thaliana* reference genome (TAIR 10.1) using HISAT2 version 2.1.0, a Bowtie2 aligner. After mapping, StringTie version 2.1.3b was employed for transcript assembly. Expression profiles for transcripts in all samples were calculated as the read count, FPKM (fragments per kilobase of transcript per million mapped reads), and TPM (transcripts per kilobase million).

### 4.3. Differential Expression Analysis and Functional Annotation

To detect transcripts expressed in response to POT1 inoculation under Pi-deficient conditions, DEG (differentially expressed gene) analysis was performed on pairwise comparisons for root and shoot samples using DESeq2. DESeq2 was preferred as it performed specific estimate variance–mean tests, and it used a model based on the negative binomial distribution to estimate the differential gene expression. The read count data were normalized with the relative log expression (RLE) method to minimize differences between samples for genes/transcripts in low expression. The RLE normalized count was adopted for a negative binomial Wald test (nbinomWaldTest) in DESeq2. Genes that satisfied the conditions of |fc| >= 2 and a negative binomial Wald Test (nbinomWaldTest) raw *p*-value < 0.05 in at least one of the comparison pairs were considered as DEGs. Venn diagrams were plotted using the jvenn tool (http://jvenn.toulouse.inra.fr/app/index.html (accessed on 12 June 2023)) to determine the differential co-expression in *A. thaliana* inoculated with POT1 under different conditions. For DEGs, gene-set enrichment analysis was performed based on gene ontology using g:Profiler tool (https://biit.cs.ut.ee/gprofiler/ (accessed on 20 June 2023)) and the KEGG database (http://www.genome.jp/kegg/ (accessed on 22 June 2023)).

### 4.4. Quantitative PCR Analysis

Quantitative real-time PCR analysis of candidate DEGs genes from the study was performed to validate the RNA-seq results. Primers were designed using NCBI primer-BLAST and are given in Table S12. Total RNA was extracted from the shoots and roots samples using a FavorPrep^™^ plant total RNA mini kit (Favorgen, Ping-Tung, Taiwan) followed by cDNA synthesis from 1 μg of total RNA using an iScript cDNA synthesis kit (Bio-Rad Laboratories, Hercules, CA, USA). Quantitative real-time PCR was then carried out on a CFX96 Real-Time system (Bio-Rad Laboratories, Hercules, CA, USA) using a 20 μL reaction volume. The reaction mixture comprised 10 ng of template cDNA, 10 μL of SsoAdvanced Universal SYBR Green Supermix (Bio-Rad Laboratories, Hercules, CA, USA), and 0.5 μM each of forward and reverse primers. The amplification conditions included an initial denaturation at 95 °C for 30 s, followed by 40 cycles of denaturation at 95 °C for 5 s and annealing at 60 °C for 30 s. The relative expression levels were calculated with the 2^−ΔΔCt^ method using actin as the reference gene to normalize the expression of target genes.

### 4.5. Proteome Analysis

Protein extraction was performed using SDT lysis buffer containing 100 mM Nacl and 1/100 volume of DTT. In short, the samples were powdered using liquid nitrogen and lysed with lysis buffer by ultrasonication for 5 min and incubation at 95 °C for 8–15 min. After centrifugation, the supernatant was alkylated with iodoacetamide for 1 h at room temperature under dark. Excess iodoacetamide and other contaminants were removed by acetone precipitation at −20 °C for 2 h. The protein pellet was dissolved in Dissolution Buffer (8 M Urea, 100 mM triethylammonium bicarbonate, pH 8.5) after being washed in cold acetone, followed by determining the protein content by Bradford assay using bovine serum albumin (BSA) as a reference. The samples were then digested with trypsin and desalted using C18 desalting column.

Quantitative proteomics analysis was performed using a Vanquish Neo upgraded UHPLC system (Thermo Fisher Scientific, San Jose, CA, USA) coupled to a Thermo Fisher Orbitrap Astral mass spectrometer with an Easy-spray (ESI) ion source. The UHPLC system was used with a C18 pre-column of 174,500 (5 mm × 300 μm, 5 μm) heated at 50 °C in a column oven and a C18 analytical column of ES906 (PepMapTM Neo UHPLC 150 µm × 15 cm, 2 μm). Mobile phase A was composed of 100% water and 0.1% formic acid, whereas mobile phase B was 80% acetonitrile and 0.1% formic acid. In the mass spectrometer, the ion spray voltage was set to 1.9 kV, the ion transfer tube temperature was 290 °C, and the mass spectrum was in a data-dependent acquisition mode with a full first-stage MS scan range of *m*/*z* 380–980. The maximum injection time was 3 ms, the primary MS resolution was 240,000 at *m*/*z* 200, and the secondary *m*/*z* acquisition range was 150 to 2000.

The *A. thaliana* Uniprot protein database containing 136,341 sequences was used as the reference database. The DIA data were imported into the DIA-NN software version 1.8.1 to extract ion pair chromatographic peaks, perform sub-ion matching, and calculate peak areas, achieving simultaneous qualitative and quantitative analyses of peptides. The library search parameters were as follows: a mass tolerance of 10 ppm for precursor ions and 0.02 Da for fragment ions. Alkylation modification of cysteine was set as a fixed modification, whereas methionine oxidation and N-terminal acetylation were included as variable modifications. DIA-NN software further filtered the search results by retaining only credible peptide spectrum matches (PSMs) with a confidence level of 99% or higher. The GO, KEGG, and COG databases were employed to annotate the identified proteins, and the proteins exhibiting a fold change greater than 1.5 and less than 0.67 with a *p* value of <0.05 were considered significantly differentially expressed for upregulated and downregulated proteins, respectively. Functional annotation clustering of the top 50 differentially expressed proteins (DEPs) was performed using the DAVID tool (https://david.ncifcrf.gov/ (accessed on 12 September 2024)).

### 4.6. Elemental Profiling

For elemental analysis, *A. thaliana* Col-0 seeds were germinated on media containing Murashige and Skoog salts, 0.25 mM MES, 10 g/L sucrose, and 0.8% agar and grown at 22 °C. After 10 days, the seedlings were transplanted into vermiculite precultured with POT1 mycelia (0.5 g) for 7 days and then cultivated under low P with 0.01 mM of KH_2_PO_4_. Simultaneously, 10-day-old seedlings were transplanted into vermiculite drenched with full P (0.50 mM of KH_2_PO_4_) and low P (0.01 mM of KH_2_PO_4_) solutions without fungal treatment. After 2 weeks of plant growth, the shoot samples were excised and dried at 60 °C for 24 h. The dried samples were ground into fine powders, and 50 mg of the powdered samples comprising three biological replicates was used for analysis. Elements such as carbon (C), hydrogen (H), nitrogen (N), and sulfur (S) were analyzed using an organic element analyzer. Other elements, viz., phosphorus (P), molybdenum (Mo), iron (Fe), potassium (K), boron (B), calcium (Ca), cobalt (Co), copper (Cu), magnesium (Mg), manganese (Mn), and sodium (Na), were determined using Agilent 7700s inductively coupled plasma mass spectrometry (ICP-MS) system (Agilent Technologies, Santa Clara, CA, USA).

### 4.7. Statistical Analysis

Statistical analysis was performed using GraphPad Prism software version 9.5 for Windows (GraphPad Software, San Diego, CA, USA). A Student’s *t*-test was used to assess the data in replicates, and an unpaired two-tailed *t*-test was used to determine significant differences across treatments at *p* < 0.05.

## 5. Conclusions

The present study provided insights into POT1-mediated growth promotion under phosphate-deprived conditions. Gene expression and proteome profiling of POT1-treated plants revealed the substantial role of the fungus in regulating numerous metabolic pathways related to growth and development in *A. thaliana*. Overall, it can be concluded that POT1 enhances plant growth by alleviating stress responses through phosphate solubilization. Our study provides valuable insights into the role of POT1 in *A. thaliana*; however, further research is essential to evaluate its effectiveness in a broader range of crops under diverse field conditions and in synergy with other beneficial organisms. Additionally, understanding the interactions and gene regulations between POT1 and other biofertilizers or soil microbes could offer new strategies for integrated agricultural practices. The application of multi-omics approaches is crucial in this context. By integrating data from genomics, transcriptomics, epigenomics, and proteomics, multi-omics provides a comprehensive understanding of gene expression and interactions at various biological levels. This holistic approach can uncover deeper insights into how POT1 functions within complex biological systems, enabling more effective strategies for crop improvement and sustainable agriculture.

## Data Availability

The raw data supporting the conclusions of this article will be made available by the authors on request.

## Supplementary Materials

The following supporting information can be downloaded at https://www.mdpi.com/article/10.3390/ijms252312865/s1.
