# Supplementary material for: Molecular Mechanisms of Phosphate Use Efficiency in Arabidopsis via Penicillium olsonii TLL1
Source: Int J Mol Sci. 2024 Nov 29;25(23):12865. doi: 10.3390/ijms252312865 (PMC11640997; doi:10.3390/ijms252312865)
Supplement: Supplementary file 1 [file ijms-25-12865-s001.zip › Supplementary Figures and Tables/RNA seq MS_Supplementary Figures.pdf]

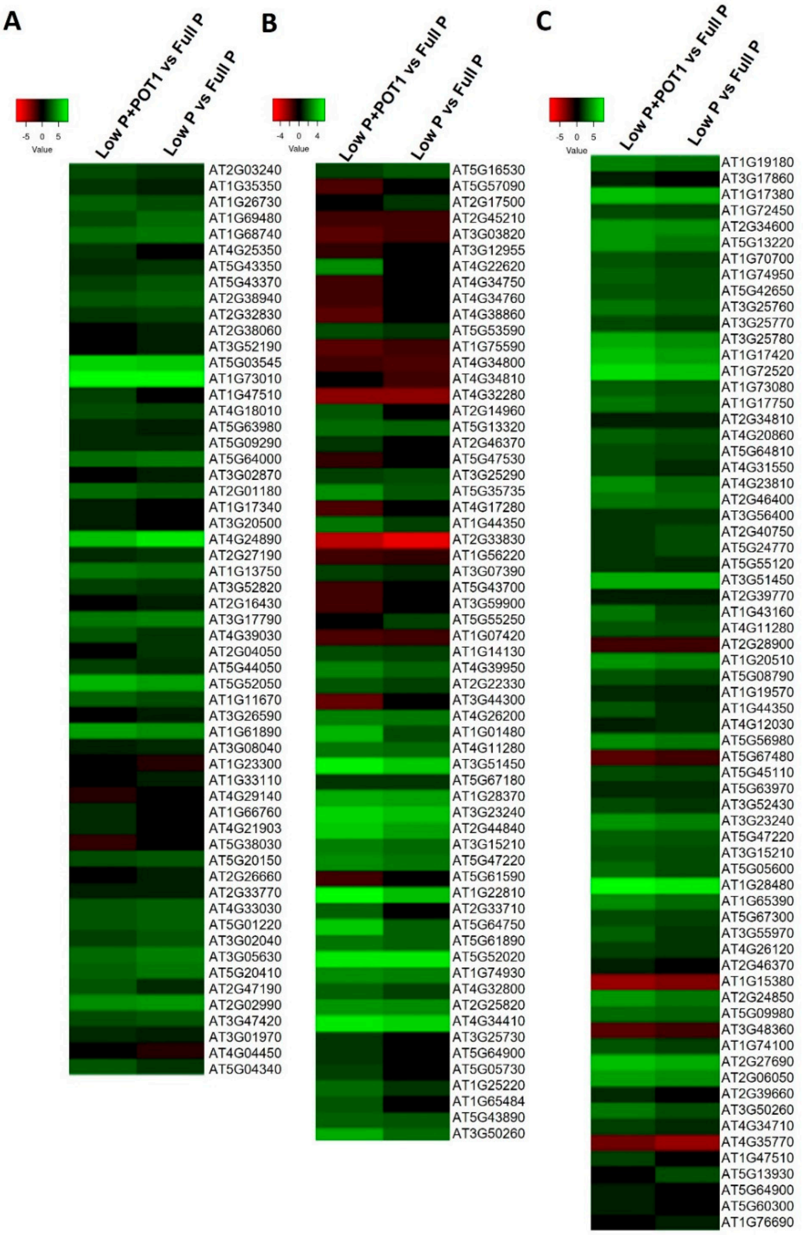

Figure S1. Heatmap of the differentially expressed genes (DEGs) in Arabidopsis treated with *Penicillium olsonii* TLL1 (POT1) under low P conditions. (**A**) DEGs related to phosphate starvation related; (**B**) auxin and ethylene related; (**C**) jasmonic acid related DEGs in the shoot of Arabidopsis treated with POT1. Heatmaps were constructed using the log2 fold change values, and the genes in green and red represent up- and down-regulated genes, respectively.

A

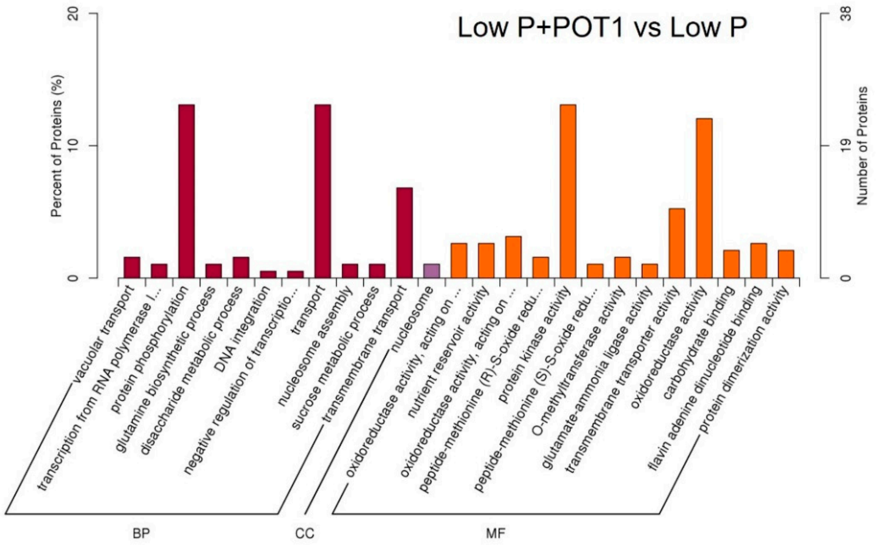

B

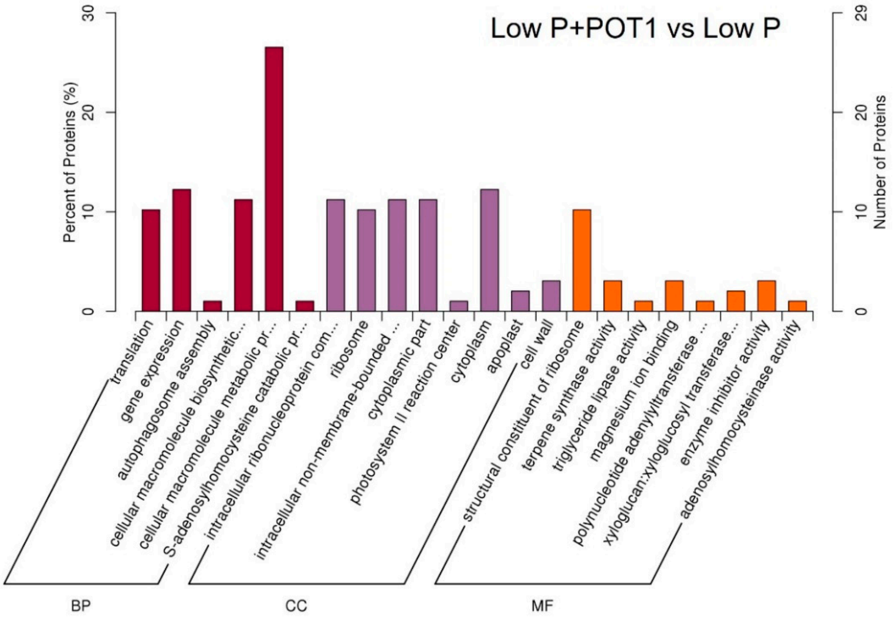

Figure S2. GO enrichment analysis of differentially expressed proteins in roots. The bar diagrams show the enrichment GO terms in (**A**) upregulated and (**B**) downregulated proteins in the three categories, biological process (BP), cellular component (CC) and molecular function (MF) for the pairwise combination, Low P+POT1 vs Low P.

A

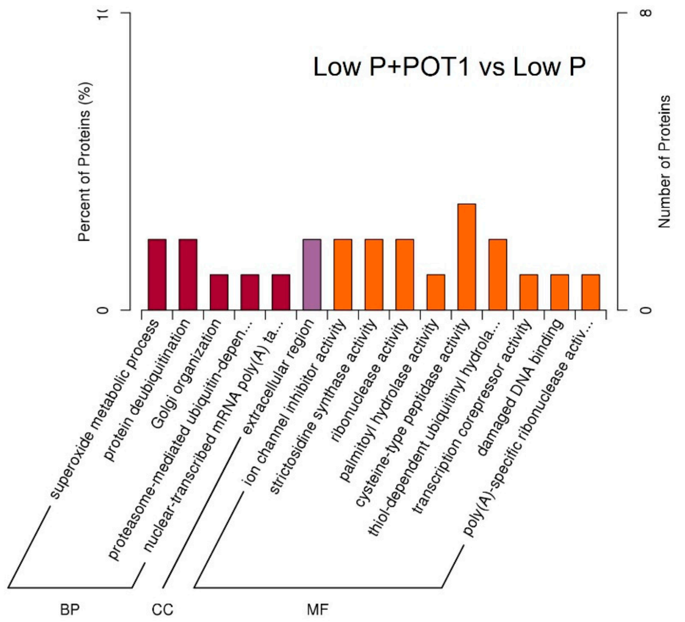

B

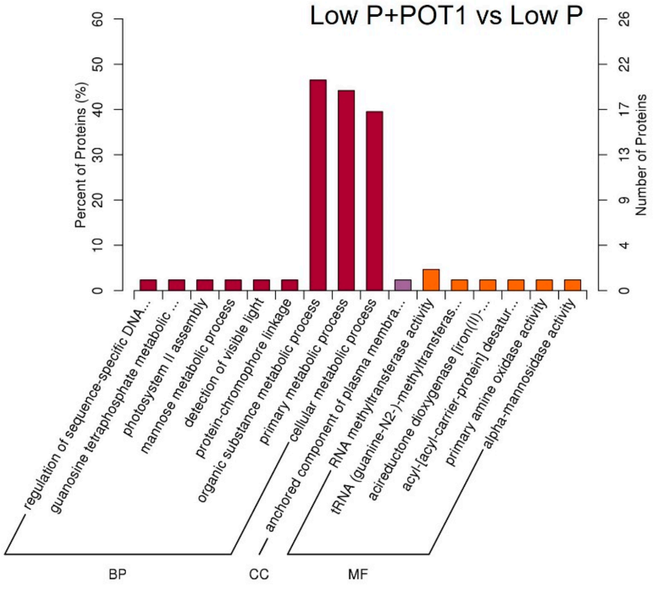

Figure S3. GO enrichment analysis of differentially expressed proteins in shoots. The bar diagrams show the enrichment GO terms in (**A**) upregulated and (**B**) downregulated proteins in the three categories, biological process (BP), cellular component (CC) and molecular function (MF) for the pairwise combination, Low P+POT1 vs Low P.

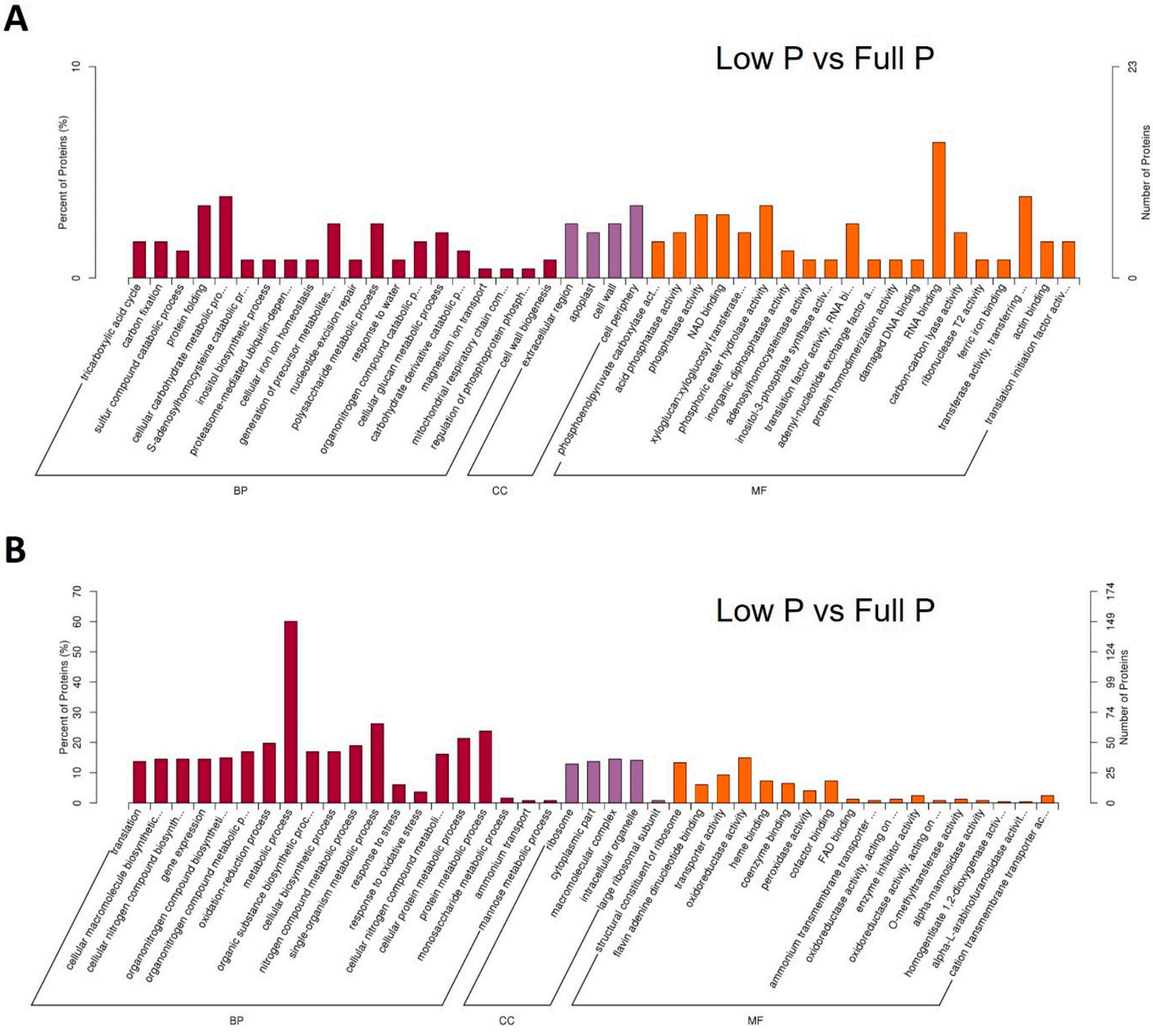

Figure S4. GO enrichment analysis of differentially expressed proteins in roots. The bar diagrams show the enrichment GO terms in (**A**) upregulated and (**B**) downregulated proteins in the three categories, biological process (BP), cellular component (CC) and molecular function (MF) for the pairwise combination, Low P vs Full P.

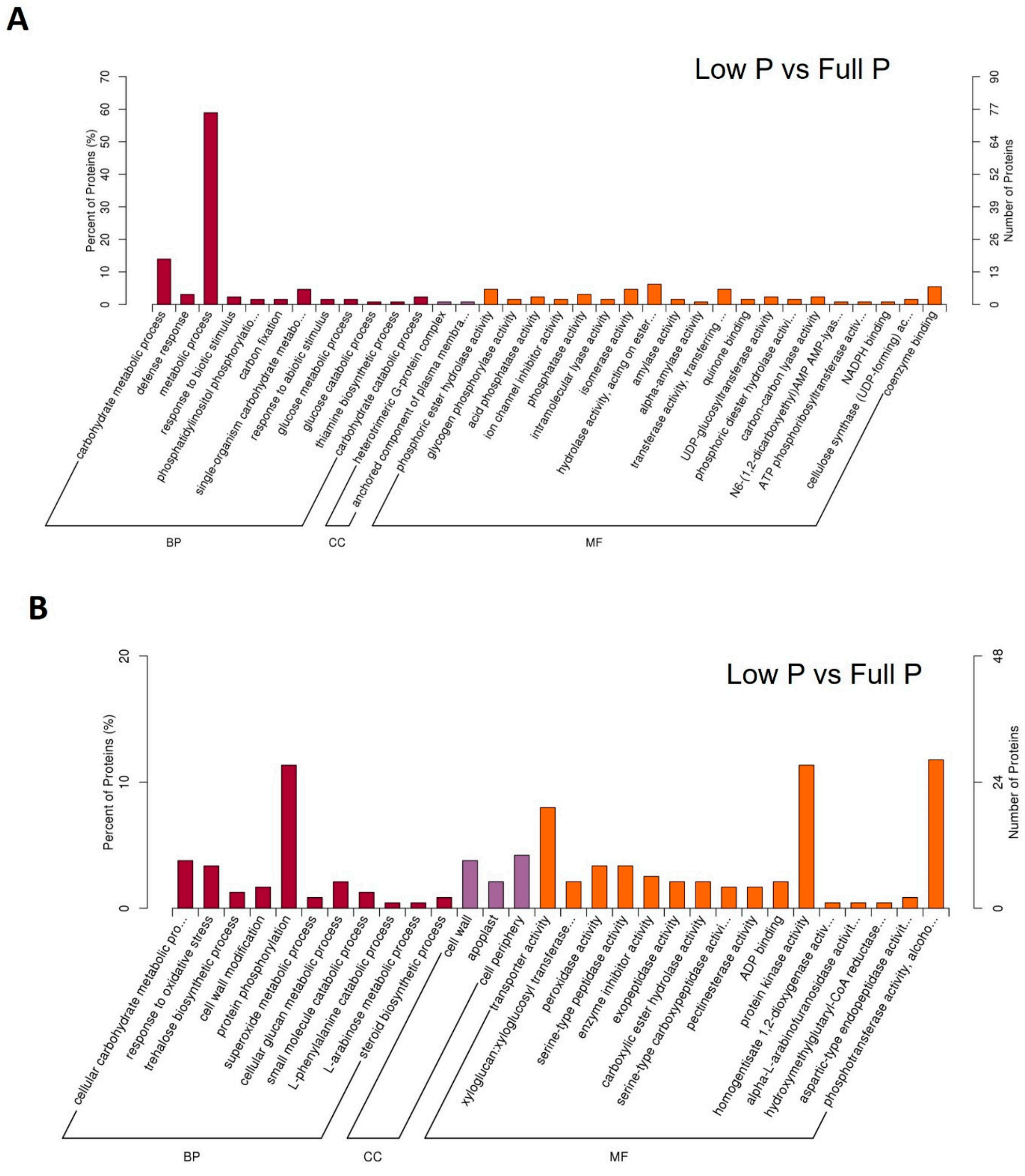

Figure S5. GO enrichment analysis of differentially expressed proteins in shoots. The bar diagrams show the enrichment GO terms in (**A**) upregulated and (**B**) downregulated proteins in the three categories, biological process (BP), cellular component (CC) and molecular function (MF) for the pairwise combination, Low P vs Full P.

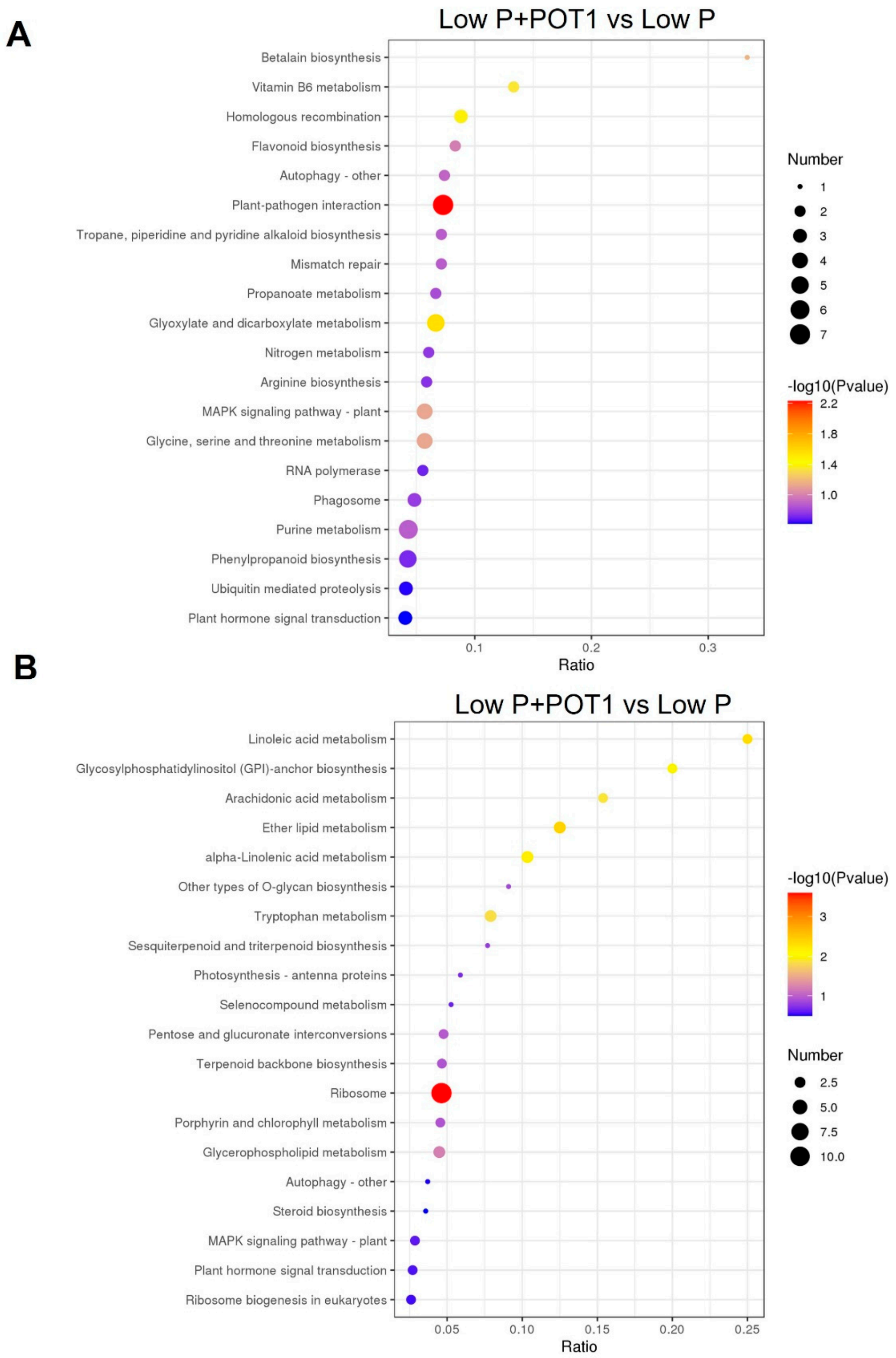

Figure S6. KEGG enrichment analysis of differentially expressed proteins in roots. The bubble plots show the significantly enriched pathways in (**A**) upregulated and (**B**) downregulated proteins in the pairwise combination, Low P+POT1 vs Low P.

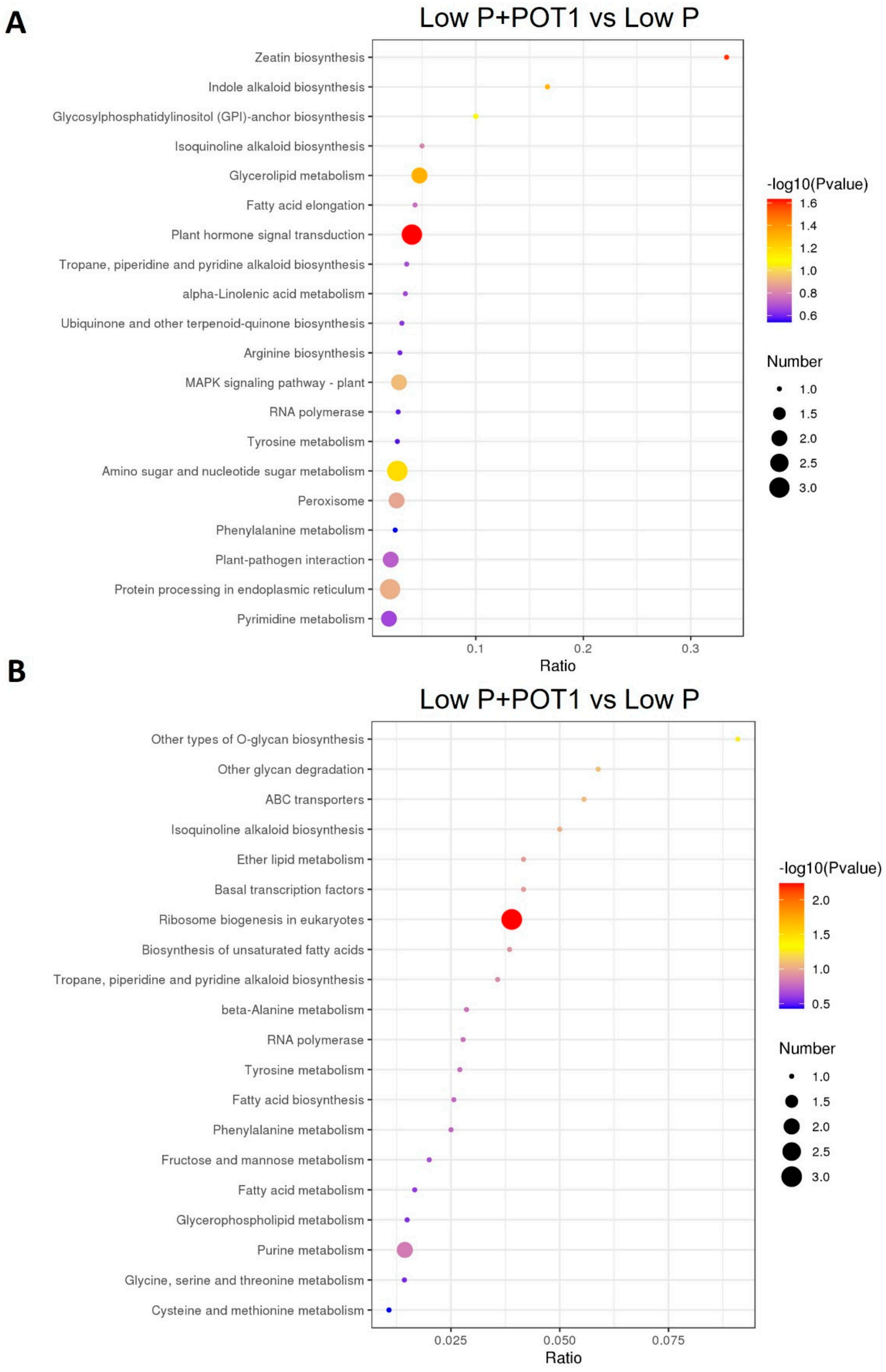

Figure S7. KEGG enrichment analysis of differentially expressed proteins in shoots. The bubble plots show the significantly enriched pathways in (**A**) upregulated and (**B**) downregulated proteins in the pairwise combination, Low P+POT1 vs Low P.

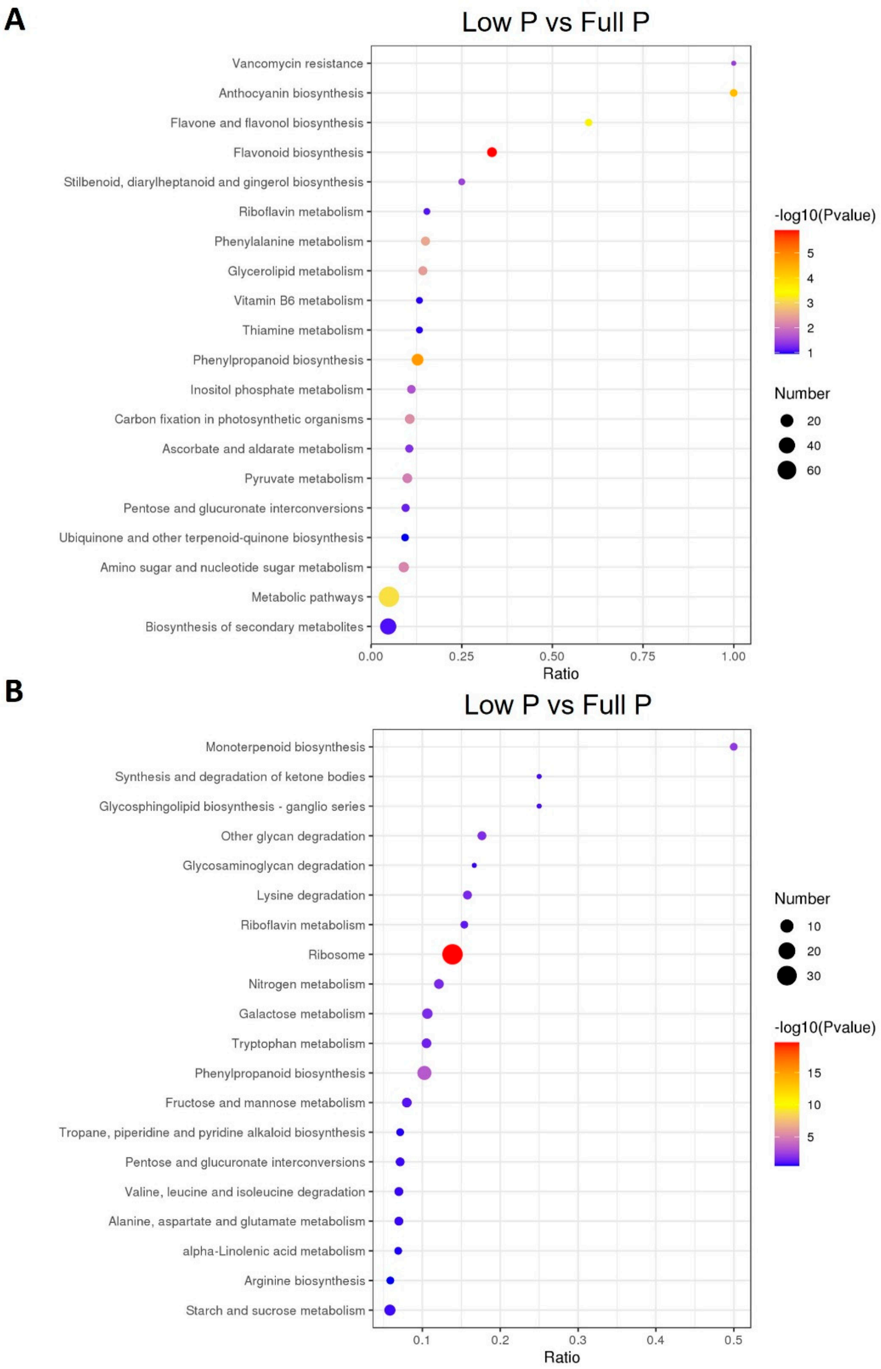

Figure S8. KEGG enrichment analysis of differentially expressed proteins in roots. The bubble plots show the significantly enriched pathways in (**A**) upregulated and (**B**) downregulated proteins in the pairwise combination, Low P vs Full P.

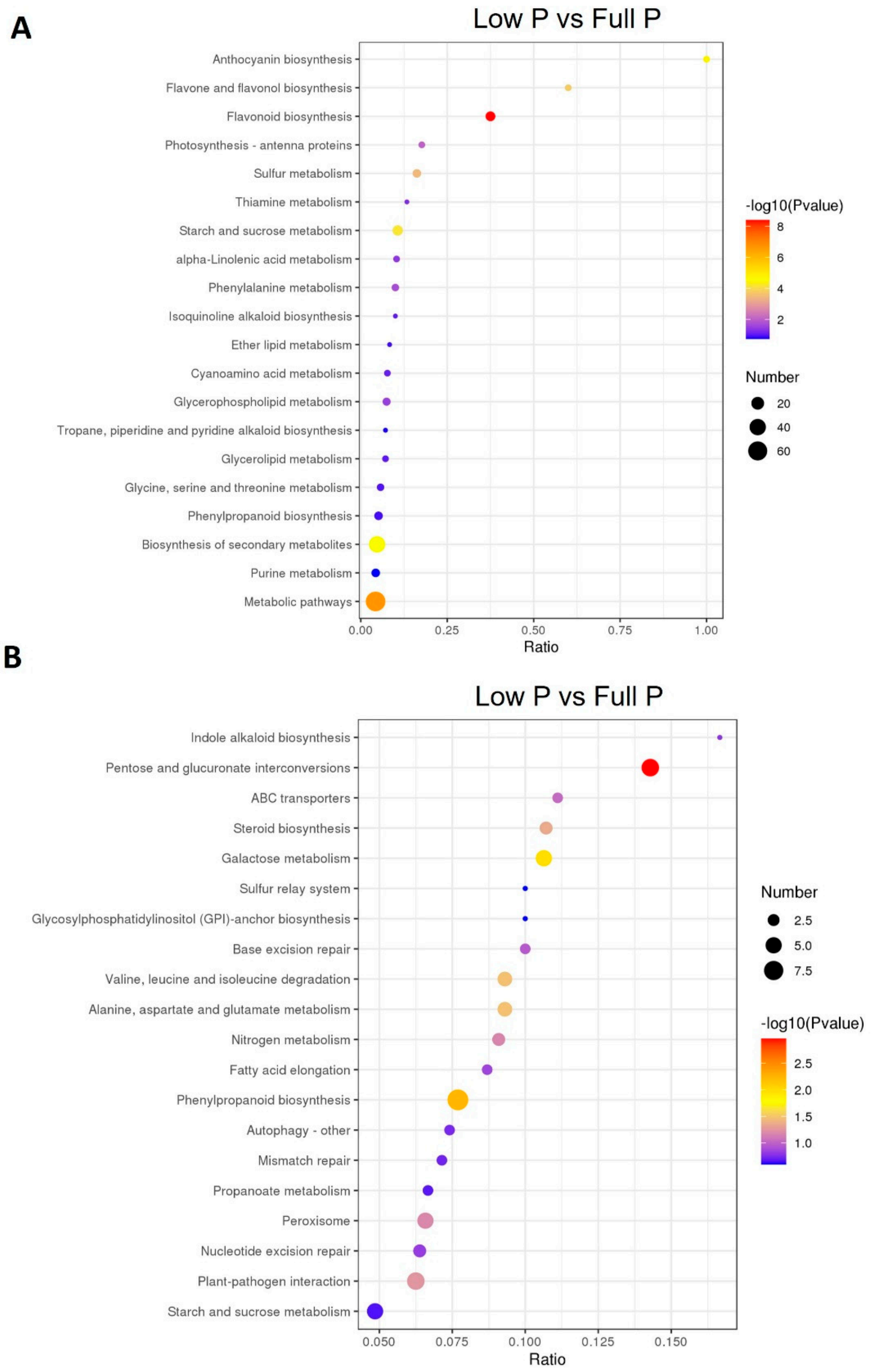

Figure S9. KEGG enrichment analysis of differentially expressed proteins in shoots. The bubble plots show the significantly enriched pathways in (**A**) upregulated and (**B**) downregulated proteins in the pairwise combination, Low P vs Full P.

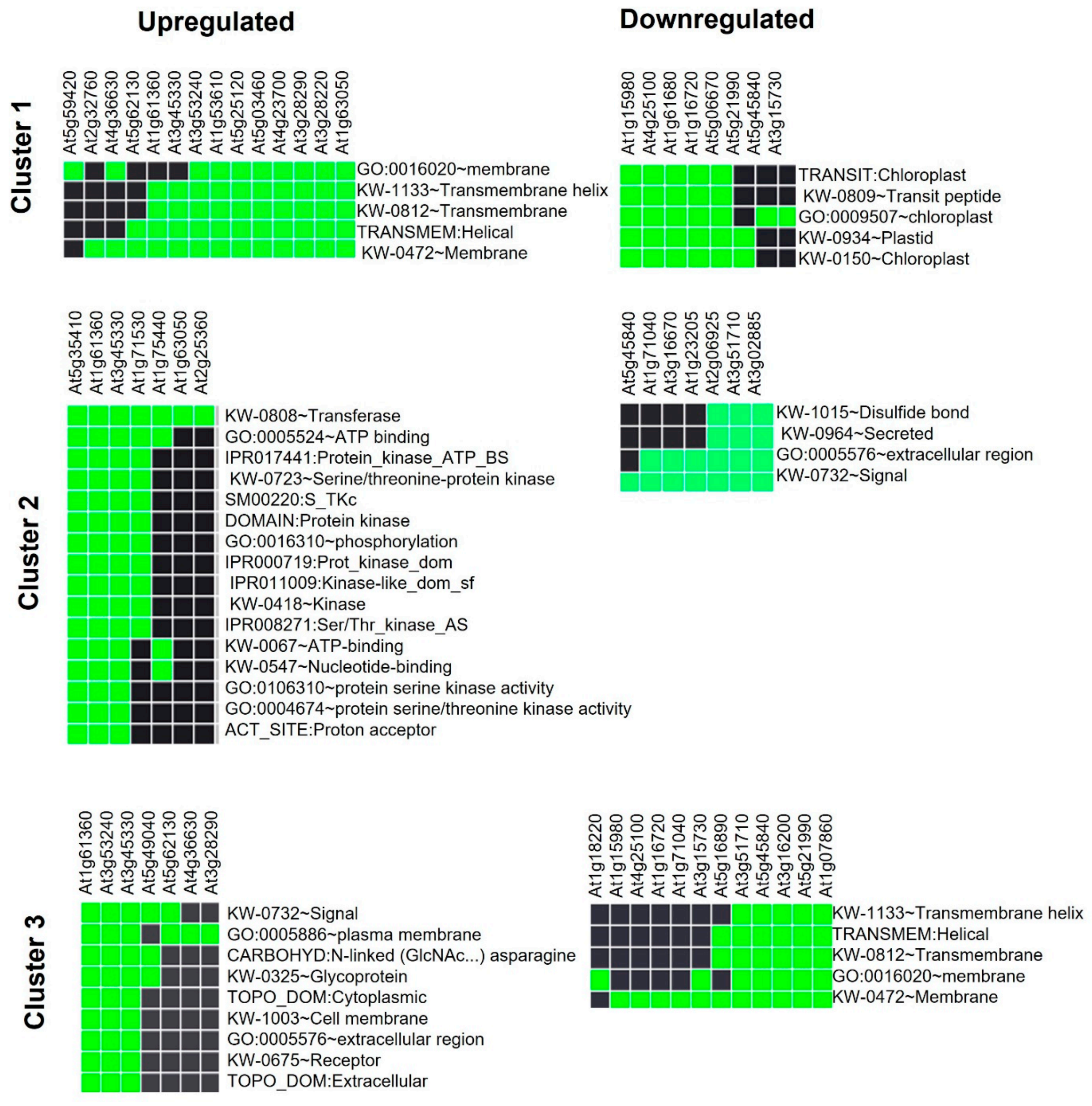

Figure S10. Functional annotation clustering of top 50 most significantly up-regulated and down-regulated proteins in roots of Low P+POT1 vs Low P. The clusters were identified using the DAVID functional annotation clustering tool with medium classification stringency. Annotated cluster represented a similarity threshold 0.50 and overlap=3, and the similarity scores ranged from high (0.75-1) to low enrichment (<0.25).

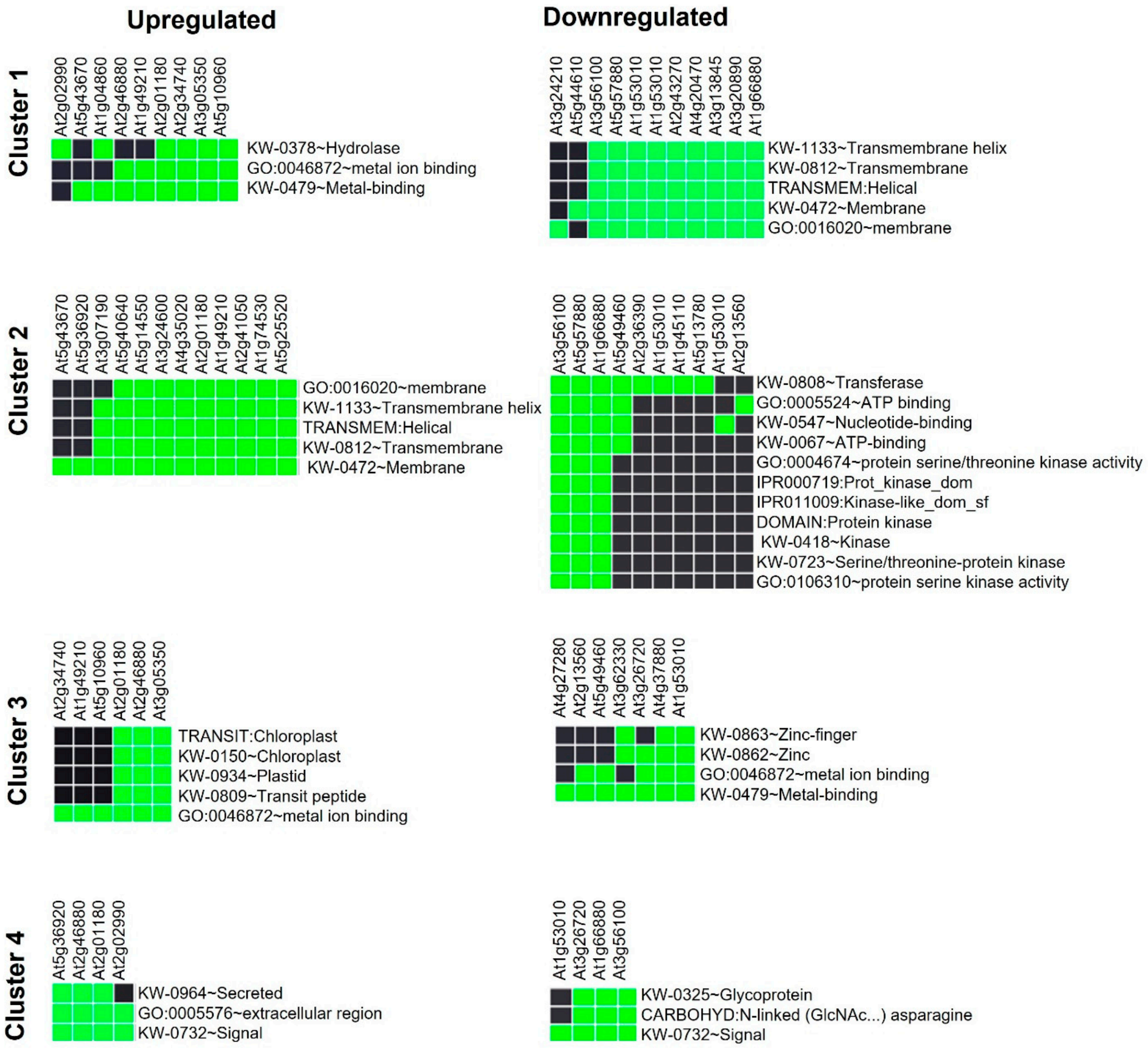

Figure S11. Functional annotation clustering of top 50 most significantly up-regulated and down-regulated proteins in shoots of Low P+POT1 vs Low P. The clusters were identified using the DAVID functional annotation clustering tool with medium classification stringency. Annotated cluster represented a similarity threshold 0.50 and overlap=3, and the similarity scores ranged from high (0.75-1) to low enrichment (<0.25).

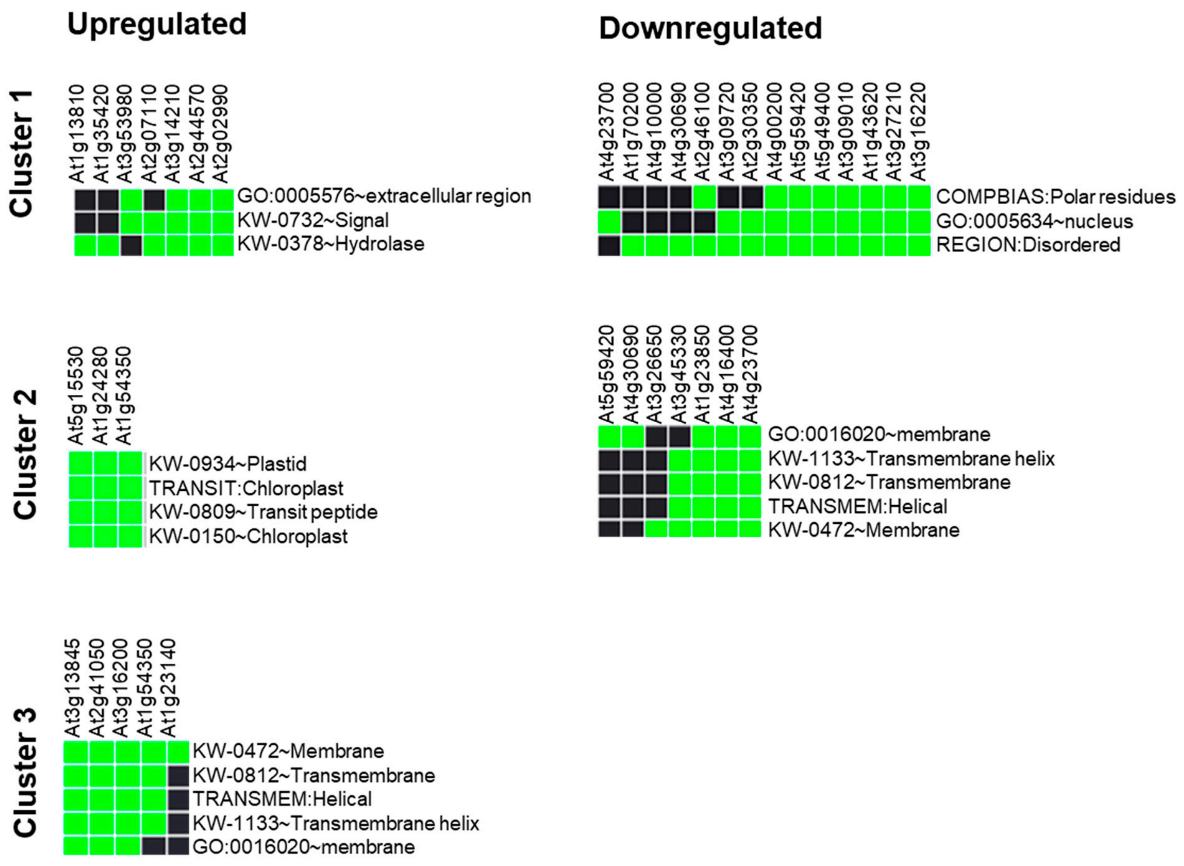

Figure S12. Functional annotation clustering of top 50 most significantly up-regulated and down-regulated proteins in roots of Low P vs Full P. The clusters were identified using the DAVID functional annotation clustering tool with medium classification stringency. Annotated cluster represented a similarity threshold 0.50 and overlap=3, and the similarity scores ranged from high (0.75-1) to low enrichment (<0.25).

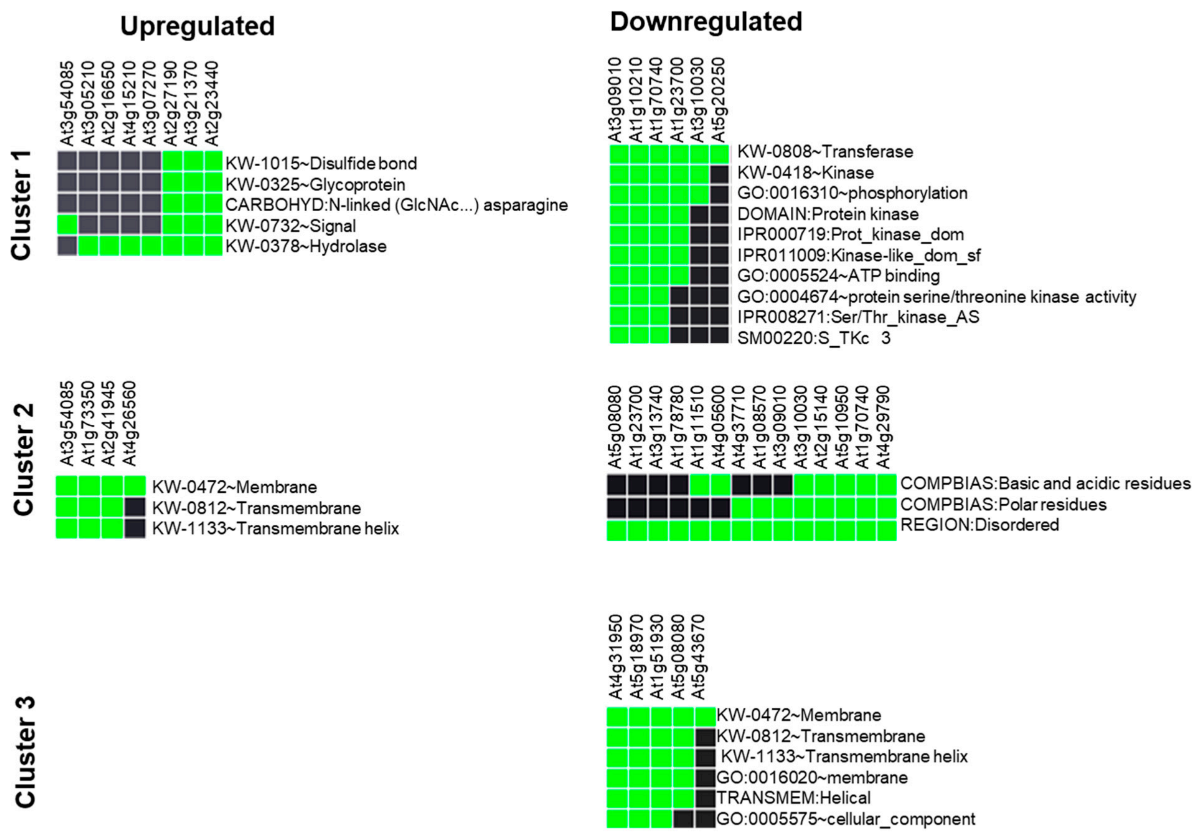

Figure S13. Functional annotation clustering of top 50 most significantly up-regulated and down-regulated proteins in shoots of Low P vs Full P. The clusters were identified using the DAVID functional annotation clustering tool with medium classification stringency. Annotated cluster represented a similarity threshold 0.50 and overlap=3, and the similarity scores ranged from high (0.75-1) to low enrichment (<0.25).
